# Tracing the metabolism of HT-2 toxin and T-2 toxin in barley by isotope-assisted untargeted screening and quantitative LC-HRMS analysis

**DOI:** 10.1007/s00216-015-8975-9

**Published:** 2015-09-03

**Authors:** Jacqueline Meng-Reiterer, Elisabeth Varga, Alexis V. Nathanail, Christoph Bueschl, Justyna Rechthaler, Susan P. McCormick, Herbert Michlmayr, Alexandra Malachová, Philipp Fruhmann, Gerhard Adam, Franz Berthiller, Marc Lemmens, Rainer Schuhmacher

**Affiliations:** Center for Analytical Chemistry, Department of Agrobiotechnology (IFA-Tulln), University of Natural Resources and Life Sciences, Vienna (BOKU), Konrad-Lorenz-Str. 20, 3430 Tulln, Austria; Institute for Biotechnology in Plant Production, IFA-Tulln, BOKU, Konrad-Lorenz-Str. 20, 3430 Tulln, Austria; Christian Doppler Laboratory for Mycotoxin Metabolism, IFA-Tulln, BOKU, Konrad-Lorenz-Str. 20, 3430 Tulln, Austria; Chemistry and Toxicology Unit, Research and Laboratory Department, Finnish Food Safety Authority (Evira), Mustialankatu 3, 00790 Helsinki, Finland; University of Applied Sciences Wr. Neustadt, Degree Programme Biotechnical Processes (FHWN-Tulln), Konrad-Lorenz-Str. 10, 3430 Tulln, Austria; Bacterial Foodborne Pathogens and Mycology Research Unit, National Center for Agricultural Utilization Research, U.S. Department of Agriculture, 1815 N. University Street, Peoria, IL 61604 USA; Department of Applied Genetics and Cell Biology, BOKU, Konrad-Lorenz-Str. 24, 3430 Tulln, Austria; Institute of Applied Synthetic Chemistry, Vienna University of Technology, Getreidemarkt 9/163, 1060 Vienna, Austria

**Keywords:** Untargeted metabolite profiling, Stable isotopic labelling, Fast polarity switching, Type A trichothecenes, Masked mycotoxins

## Abstract

An extensive study of the metabolism of the type A trichothecene mycotoxins HT-2 toxin and T-2 toxin in barley using liquid chromatography coupled to high-resolution mass spectrometry (LC-HRMS) is reported. A recently developed untargeted approach based on stable isotopic labelling, LC-Orbitrap-MS analysis with fast polarity switching and data processing by MetExtract software was combined with targeted LC-Q-TOF-MS(/MS) analysis for metabolite structure elucidation and quantification. In total, 9 HT-2 toxin and 13 T-2 toxin metabolites plus tentative isomers were detected, which were successfully annotated by calculation of elemental formulas and further LC-HRMS/MS measurements as well as partly identified with authentic standards. As a result, glucosylated forms of the toxins, malonylglucosides, and acetyl and feruloyl conjugates were elucidated. Additionally, time courses of metabolite formation and mass balances were established. For absolute quantification of those compounds for which standards were available, the method was validated by determining apparent recovery, signal suppression, or enhancement and extraction recovery. Most importantly, T-2 toxin was rapidly metabolised to HT-2 toxin and for both parent toxins HT-2 toxin-3-*O*-*β*-glucoside was identified (confirmed by authentic standard) as the main metabolite, which reached its maximum already 1 day after toxin treatment.

**Electronic supplementary material:**

The online version of this article (doi:10.1007/s00216-015-8975-9) contains supplementary material, which is available to authorized users.

**Graphical Abstract:**
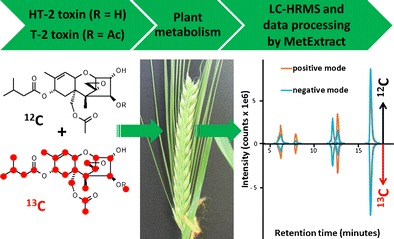
Isotope-assisted untargeted screening of HT-2 toxin and T-2 toxin metabolites in barley

## Introduction

HT-2 toxin (HT2) and T-2 toxin (T2) are secondary metabolites of fungi belonging to the genus *Fusarium* and are classified as type A trichothecene mycotoxins. Small grain cereals, oats, wheat and barley, are especially affected by type A trichothecene-producing fungi and consequently are prone to contamination with HT2 and T2 [1, 2]. The toxins differ structurally in an acetyl group at the C-4 position (see Electronic Supplementary Material (ESM) Fig. S1) but show similar toxicological effects such as immunotoxicity and haematotoxicity [3].

Plants employ various detoxification mechanisms to cope with the adverse effects of mycotoxins. For instance, phase I metabolism processes (enzymatic hydrolysis, oxidation and reduction) as well as phase II metabolism processes (covalent binding of, e.g. glucose, malonic acid, sulphuric acid, amino acids or glutathione) are used by the affected plants to inactivate xenobiotics [4]. Understanding the plant metabolism of mycotoxins and thus the resulting metabolic derivatives is becoming increasingly important for risk assessment. Up to now, there is no legislation for these so-called masked mycotoxins in food or feed, although studies [5, 6] have indicated that they might exhibit similar toxicity when cleaved during digestion [4]. Only limited knowledge exists about the biotransformation process of HT2 and T2 in plants. In an early article, Mirocha et al. [7] reported the occurrence of HT2, T-2-tetraol, 3′-hydroxy-HT-2 and 3′-hydroxy-T-2 formed in T2-treated *Baccharis* species. A few authors have described monoglucoside derivatives [2, 8–12] (HT2-Glc and T2-Glc) and diglucoside derivatives [10, 11] (HT2-di-Glc) to be formed in planta. Additionally, an extensive study of the metabolism of HT2 and T2 in wheat was recently performed in our lab [13]. Whilst the work presented here details the technical aspects of metabolite detection and characterisation, the study mentioned above focuses on the biological interpretation of HT2 and T2 metabolism in wheat and therefore complements the presented study.

Generally, the global untargeted analysis of endogenous metabolites and metabolic products of xenobiotics in biological systems constitutes a major challenge because of their chemical and physical diversity [14, 15]. Liquid chromatography coupled to high-resolution mass spectrometry (LC-HRMS) is often used for metabolism studies. To cover as many metabolites as possible, it has been shown that measurements in positive and negative polarity should be performed which is ideally combined in time-saving fast polarity switching mode [16]. However, untargeted full scan LC-HRMS measurements produce large datasets which have to be interpreted properly. To this end, bioinformatic tools are frequently applied to extract relevant signals from raw data. Data interpretation with the single use of statistical methods such as principle components analysis (PCA) is limited to differential comparison between the tested experimental conditions and is partly error-prone [14]. One way to circumvent this problem is the employment of isotopic labelling approaches. Several authors have successfully performed metabolism studies employing stable isotopically labelled tracers [17–19]. In a further step, software tools to recognise specific characteristics of labelling in measurement data enable rapid and automated data evaluation [20, 21]. Bueschl et al. [22, 23] developed a programme to extract signals of labelled metabolites from LC-MS data. This procedure allows a truly untargeted analysis with the complete removal of unwanted signals coming from biological matrix, solvents, reagent impurities, background and instrument noise.

The objective of this study was to investigate the metabolism of the two major type A trichothecenes HT2 and T2 in barley. For this purpose, an untargeted method was applied by combining stable isotopic labelling, LC-Orbitrap-MS analysis in fast polarity switching mode and MetExtract data processing. After structure annotation, quantification experiments were performed with a Q-TOF instrument in order to study the kinetics of the metabolism of parent toxins and their major biotransformation products. To the best of our knowledge, this is the first demonstration of an automated fast polarity switching approach used to study the metabolism of a xenobiotic in planta. To cover the set of potential toxin derivatives as complete as possible, measurements in both polarities were essential resulting in complementary information, revealing novel metabolites.

## Materials and methods

In general, three main experiments were carried out named hereafter qualitative screening, structure annotation and time course experiment (workflow presented in ESM Fig. S2).

### Chemicals and standards

Methanol (MeOH) and acetonitrile (ACN) were purchased from VWR (Vienna, Austria). Formic acid (FA) and Tween 20 were obtained from Sigma-Aldrich (Vienna, Austria), whilst ammonium formate solution (5 M, NH_4_HCO_2_) was provided by Agilent Technologies (Waldbronn, Germany). All solvents were LC gradient grade or higher. Purified water (H_2_O) was produced by reverse osmosis and an ELGA Purelab Ultra Mk2 Analytic system from Veolia (Vienna, Austria). The following standard compounds (all purities specified by the supplier) were purchased from Romer Labs (Tulln, Austria): crystalline non-labelled HT2 (purity 92 %) and T2 toxin (purity 85 %) as well as uniformly labelled U-^13^C_22_ HT2 (purity 86 %; 99.3 atom% ^13^C) and U-^13^C_24_ T2 (purity 98 %; 99.6 atom% ^13^C). Barley test solutions of HT2 and T2 (qualitative screening experiment) were prepared by mixing non-labelled and labelled toxins (both 2,000 mg L^−1^) 1 + 1 (*v*/*v*) to obtain a concentration of 1,000 mg L^−1^ per toxin in ACN/MeOH/H_2_O 10 + 45 + 45 (*v*/*v*/*v*). All biological material treated with this mixture is later referred to as labelled barley samples or ^12^C/^13^C samples. Time course experiments were performed with a test solution of non-labelled HT2 and T2 toxin with 1,000 mg L^−1^ in ACN/H_2_O 1 + 1 (*v*/*v*) + 1 % Tween 20. Respective samples are later referred to as non-labelled or ^12^C samples. For each experiment, test solutions solely containing the corresponding solvent mixtures (mock) were prepared to obtain blank samples. Analytical standards of HT2 and T2 toxin for quantification experiments were purchased from Romer Labs at concentrations of 100 mg L^−1^ (purity > 99.9 %) and 101 mg L^−1^ (purity > 99.9 %), respectively in ACN. Standard T2-*α*-Glc was prepared as described by McCormick et al. [12]. HT2-3-*O*-*β*-Glc and 3-acetyl-T2 were enzymatically produced or chemically synthesised within the scope of other studies (unpublished data). Highly pure standards of HT2 and T2 were used as raw materials, the final products were characterised by nuclear magnetic resonance measurements and the purities were estimated to be ≥95 %. Stock solutions of HT2-3-*O*-*β*-Glc, 3-acetyl-T2 and T2-*α*-Glc were prepared by dissolving in ACN to obtain concentrations of 1,000, 5,000 and 2,000 mg L^−1^, respectively.

### Cultivation of barley plants

For the qualitative screening and the time course experiment, barley (*Hordeum vulgare* L. sensu lato) variety ‘Calcule’ was selected. This is a two-row spring barley bred by Saatzucht Streng-Engelen GmbH & Co. KG (Germany). Seeds of the barley variety were germinated. Pots (diameter 23 cm) were filled with 7-L portions of a homemade substrate (mix of 500 L heat-sterilised compost, 250 L peat, 10 kg sand and 250 g rock flour). In each pot, five seedlings were planted. The experimental design was a completely randomised block with three biological replications (treatment of three individual barley ears per treatment group) for both plant experiments.

During the whole experiment, the pots were watered if required (typically three times per week). Plants for qualitative screening were grown in the greenhouse and after tillering transferred to a growth chamber with computer-controlled settings for light, temperature and relative air humidity. Light intensity was 560 μmol s^−1^ m^−2^ at 1 m above the soil. Relative air humidity was set between 60 and 70 % during plant growth. Temperature (day/night) and duration of illumination (hours) varied according to the development stage of the plants: after planting until the end of tillering, 12 °C/10 °C/12 h; end tillering until mid-stem extension when the ear starts to swell, 14 °C/10 °C/14 h; mid-stem extension to start heading, 16 °C/14 °C/14 h; from the start of heading until start of flowering, 18 °C/14 °C/14 h; and from the start of flowering until the end of the experiments including application of the test solutions and sampling, 20 °C/18 °C/16 h.

The time course experiment was carried out exactly as described above with the following modifications: after tillering the plants remained in the greenhouse with computer-controlled settings for light, temperature and relative air humidity. Light intensity was 370 μmol s^−1^ m^−2^ at 1 m above the soil (measured after sunset).

### Treatment and harvest of barley plants

Experiments included three treatment groups, HT2, T2 and mock, which were applied separately on different barley ears in triplicate. Treatment started at flowering stage of the respective ears. Test solutions were injected into the spikelets with an electronic pipette. To enhance toxin diffusion into the plants, small transparent plastic bags were internally wetted by spraying with purified water, placed over the barley ears after each treatment step and removed 24 h (±2 h) later. On the day of harvest, treated barley ears were cut, weighed, immediately frozen in liquid nitrogen and stored at −80 °C until further processing.

Treatment for the qualitative screening experiment was based on pipetting 5 μL of the test solution (mixture of non-labelled and labelled toxins and mock) into spikelets of barley ears. The lowest four spikelets per ear (two per row) were treated, and 2, 4 and 5 days later, we continued with the next four spikelets above. Finally, on the sixth day, two more adjacent spikelets were treated resulting in a total of 18 treated spikelets or 90 μg applied toxin (native and labelled toxin). Treated plants were harvested on the seventh day resulting in a total of five time points per ear (treatment 7, 5, 3, 2 and 1 days before harvest) per treatment group. One extra time point was monitored in the form of a single treatment per treatment group. This was performed by treating 20 spikelets (two per row) of one ear with a total amount of 100 μg toxin and harvesting at full-ripening stage (approx. 8 weeks after treatment). Concerning all previously mentioned treatments, only treated parts of the ears were analysed.

The following treatment procedure was used for the time course experiments of HT2 and T2: For each toxin plus mock, in total, 20 spikelets (two per row) were treated with 10 μL of the test solution (non-labelled toxins 1,000 mg L^−1^ and mock) on a single day. This resulted in 200 μg applied toxin per ear. Whole ears were sampled immediately (time point 0), 1, 3 and 7 days after treatment and at full-ripening stage (approx. 6 weeks after treatment).

### Sample preparation

Frozen barley ears were milled with a ball mill (MM 301 Retsch, Haan, Germany) for 30 s at 30 Hz under cooled conditions (liquid nitrogen). Milled samples were weighed (100 ± 2 mg) into Eppendorf tubes. Samples were extracted by adding 500 μL ACN/H_2_O/FA 79 + 20.9 + 0.1 (*v*/*v*/*v*), vortexing for 10 s and shaking on a rotary shaker in horizontal position (GFL 3017, Burgwedel, Germany) at room temperature for 90 min with 200 rpm. After centrifugation for 10 min at 22,570×*g*, supernatants were transferred to HPLC vials. Raw extracts of labelled samples plus mock samples were diluted 1 + 3 (*v*/*v*) with 0.1 % aqueous FA prior to the LC-Orbitrap-MS measurement. For each treatment group, one extract was additionally measured undiluted as well as concentrated by a factor of 4 (evaporated to dryness and dissolved in 1/4 volume). For structure elucidation, LC-Q-TOF-MS/MS spectra were recorded with undiluted raw extracts of ^12^C/^13^C, as well as ^12^C (time course experiment) samples. Quantification of HT2, T2 and its metabolites was performed with ^12^C sample extracts which were partially measured undiluted but also diluted 1 + 9 (*v*/*v*) and 1 + 49 (*v*/*v*) with ACN/H_2_O 1 + 1 (*v*/*v*).

### LC-HRMS(/MS) analysis

#### Qualitative screening

Labelled samples and mock samples were measured with an UltiMate 3000 HPLC system coupled to an Exactive Plus Orbitrap mass spectrometer (both from Thermo Fisher Scientific). Ten microlitres of sample solution was injected into the system. For chromatographic separation, a Zorbax SB-C18 column (150 × 2.1 mm, 3.5 μm; Agilent Technologies) was used at 25 °C and at a flow rate of 250 μL min^−1^. Mobile phases consisted of H_2_O (eluent A) and MeOH (eluent B), both containing 0.1 % FA (*v*/*v*) and 5 mM NH_4_HCO_2_. Gradient method 1 was as follows: 0–0.5 min, 10 % B; 0.5–20.0 min, 10–100 % B; 20.0–25.0 min, 100 % B; 25.0–25.1 min, 100–10 % B; and 25.1–30.0 min, 10 % B. Mass spectrometric analysis was performed in fast polarity switching mode using electrospray ionisation. Applied settings were similar to Kluger et al. [16] with some modifications: Automatic gain control was set to 5 × 10^5^, and a maximum injection time of 500 ms was used. Full scan measurement was carried out in the scan range of *m*/*z* 130–1,300 with a resolution of 70,000 full width at half maximum (FWHM) at *m*/*z* 200. The instrument was calibrated with Pierce Ion Calibration Solution in both polarity modes prior to analysis. For data evaluation, software Thermo Xcalibur 2.2 was applied.

#### Structure annotation and time course experiment

Further qualitative and quantitative measurements were performed with a 1290 Infinity UHPLC system coupled to a 6550 iFunnel Q-TOF mass spectrometer (Agilent Technologies). The chromatographic method employed a Zorbax SB-C18 Rapid Resolution HD column (150 × 2.1 mm, 1.8 μm; Agilent Technologies), at 30 °C and at a flow rate of 250 μL min^−1^. The same mobile phase composition was used as above described for Orbitrap measurements. For the acquisition of LC-HRMS/MS spectra, gradient method 2 was applied: 0–0.5 min, 10 % B; 0.5–20.0 min, 10–100 % B; 20.0–22.0 min, 100 % B; 22.0–22.1 min, 100–10 % B; and 22.1–25.0 min, 10 % B. To differentiate between isomers of formed toxin metabolites in the chromatogram and to compare with available standards, the longer gradient method 3 was used: 0–0.5 min, 20 % B; 0.5–3.0 min, 20–45 % B; 3.0–43.0 min, 45–65 % B; 43.0–43.1 min, 65–100 % B; 43.1–45 min, 100 % B; 45.0–45.1 min, 100–20 % B; and 45.1–50.0 min, 20 % B. Relative and absolute quantification experiments (time course experiments) were performed with a shortened gradient method 4: 0–0.5 min, 20 % B; 0.5–6.0 min, 20–100 % B; 6.0–8.0 min, 100 % B; 8.0–8.1 min, 100–20 % B; and 8.1–10.0 min, 20 % B.

LC-Q-TOF full scan mass spectra and LC-MS/MS spectra were acquired with 2 GHz in positive and negative mode within *m*/*z* 50–1,500 at a scan rate of 3 spectra s^−1^. For all measurements, the following MS settings were used: capillary voltage, 4,000 V; nozzle voltage, 500 V; fragmentor voltage, 380 V; drying gas temperature and flow, 130 °C and 14 L min^−1^, respectively; nebulizer, 30 psig; and sheath gas temperature and flow, 300 °C and 10 L min^−1^, respectively. Precursor ion selection for fragmentation occurred in the quadrupole with an isolation width of *m*/*z* 1.3. Mass accuracy of Q-TOF instrument was checked and potentially optimised before analysis. Continually infused reference masses (positive *m*/*z* 121.0509, *m*/*z* 922.0098; negative *m*/*z* 112.9856, *m*/*z* 966.0007) were used for internal mass calibration during the measurement. Data were acquired with MassHunter Acquisition software B.05.01, and data evaluation was performed with MassHunter Qualitative and Quantitative Analysis B.06.00.

### Data processing by MetExtract

Acquired profile mode data were centroided and converted to the mzXML data format [24] with ProteoWizard [25] and successively processed with the in-house developed software MetExtract [16]. The tool first searched for characteristic isotope patterns of native (^12^C or *M* and *M* + 1) and partially ^13^C-labelled (^13^C or *M′* and *M′* − 1) metabolite ions (ion pairs) in each MS scan. The observed *m*/*z* difference between ^12^C/^13^C metabolite ion pairs corresponded to *n*-labelled carbon atoms originating from the uniformly ^13^C-labelled tracer. This value had to show less than ±4 ppm deviation (based on preliminary evaluation of the mass accuracy from raw data) from the theoretical *m*/*z* difference (Δ*m*/*z* = *n* × 1.00335/charge). Moreover, the observed ratio of the ^12^C/^13^C ion pairs had to be approximately 1 (±0.5). Extracted ion chromatograms (EICs, *m*/*z* extraction window of ±5 ppm) generated for *M* and *M*′ ions were recognised as chromatographic peaks with the algorithm of Du et al. [26] and had to show a minimum Pearson correlation of 0.75. Such extracted feature pairs were then convoluted into feature groups (i.e. metabolites) using a minimum Pearson correlation of 0.9.

### Method validation for quantification

For in-house method validation, apparent recovery (*R*_A_), signal suppression or enhancement (SSE, also known as matrix effects) and extraction recovery (*R*_E_) were determined according to Sulyok et al. [27]. Two different barley blank matrices were used for this purpose: mock time point 1 day and mock time point ripen. A 1-mg L^−1^ (per toxin) stock solution including HT2, T2, HT2-3-*O*-*β*-Glc, 3-acetyl-T2 and T2-*α*-Glc in ACN was prepared. Blank samples were spiked at one level to obtain 300 μg L^−1^ per toxin in final matrix solution and were analysed in biological triplicate. Apparent recovery was evaluated by spiking stock solution before extraction to milled mock samples. Solvent was evaporated overnight, and extraction was conducted on the next day according to the procedure mentioned above. Matrix effects were determined by adding stock solution after extraction of blank mock samples to obtain 300 μg L^−1^ per toxin in undiluted matrix solution as well as in 1 + 9 (*v*/*v*) and 1 + 49 (*v*/*v*) dilutions (diluted with ACN/H_2_O 1 + 1 (*v*/*v*)). After LC-Q-TOF-MS measurement, EICs of the target analytes ([M + NH_4_]^+^ adducts) were automatically extracted by MassHunter Quantitative Analysis software with a *m*/*z* extraction window of ±30 ppm (based on preliminary evaluation of the mass accuracy from raw data). *R*_A_ and SSE were provided by dividing the peak area of the respective metabolite obtained for spiked matrix sample by the area of a corresponding standard (mean value, derived from triplicate) and multiplying by the factor of 100. Extraction recovery was calculated by using the ratio of *R*_A_ to SSE. Mean values and relative standard deviations (RSDs) were calculated from *R*_A_, SSE and *R*_E_ in triple determination.

### Time course of metabolite formation—absolute and relative quantification

Absolute and relative quantification was performed with the UHPLC-Q-TOF instrument in positive full scan mode with chromatographic gradient method 4 and was based on EICs of ammonium adducts of the respective analytes (*m*/*z* extraction window of ±30 ppm). Where standards were available, absolute amounts of HT2, T2 and its metabolites could be plotted versus harvest time point after treatment. The following compounds were quantified: HT2, T2, HT2-3-*O*-*β*-Glc, 3-acetyl-T2 and T2-Glc. Although the T2 metabolite T2-Glc was annotated as T2-*β*-Glc, available standard T2-*α*-Glc was used for quantification assuming similar ionisation efficiency. External calibration was applied with concentrations at six levels in the range of 3–1,000 μg L^−1^, and linear calibration curves were 1/*x* weighted. Biological replicates and different dilutions (mentioned above) of non-labelled samples were analysed. Metabolite levels in respective matrices corresponding to a signal-to-noise (*S*/*N*) ratio of 10 served as limit of quantification (LOQ). Concentration values were multiplied by ear weight to obtain results in microgram/ear or subsequently in micromole/ear, respectively. Based on the estimated method precision, matrix effects were only corrected if below 85 % and above 115 %. For other biotransformation products, relative quantification was carried out by integrating peaks of EICs above a *S*/*N* of 3 in matrix (limit of detection, LOD) and performing normalisation by ear weight. Thus, time courses of normalised metabolite peak areas were graphically displayed. Each time point value was presented as mean value ± standard deviation (*n* = 3).

## Results and discussion

The qualitative screening of HT2 and T2 metabolites in barley was generally based on treatment of barley ears with a mixture of non-labelled and uniformly ^13^C-labelled toxin, extraction, measurement of sample extracts with LC-Orbitrap-MS in fast polarity switching mode and data processing by MetExtract.

### HT2 metabolism in barley

**Fig. 1:**
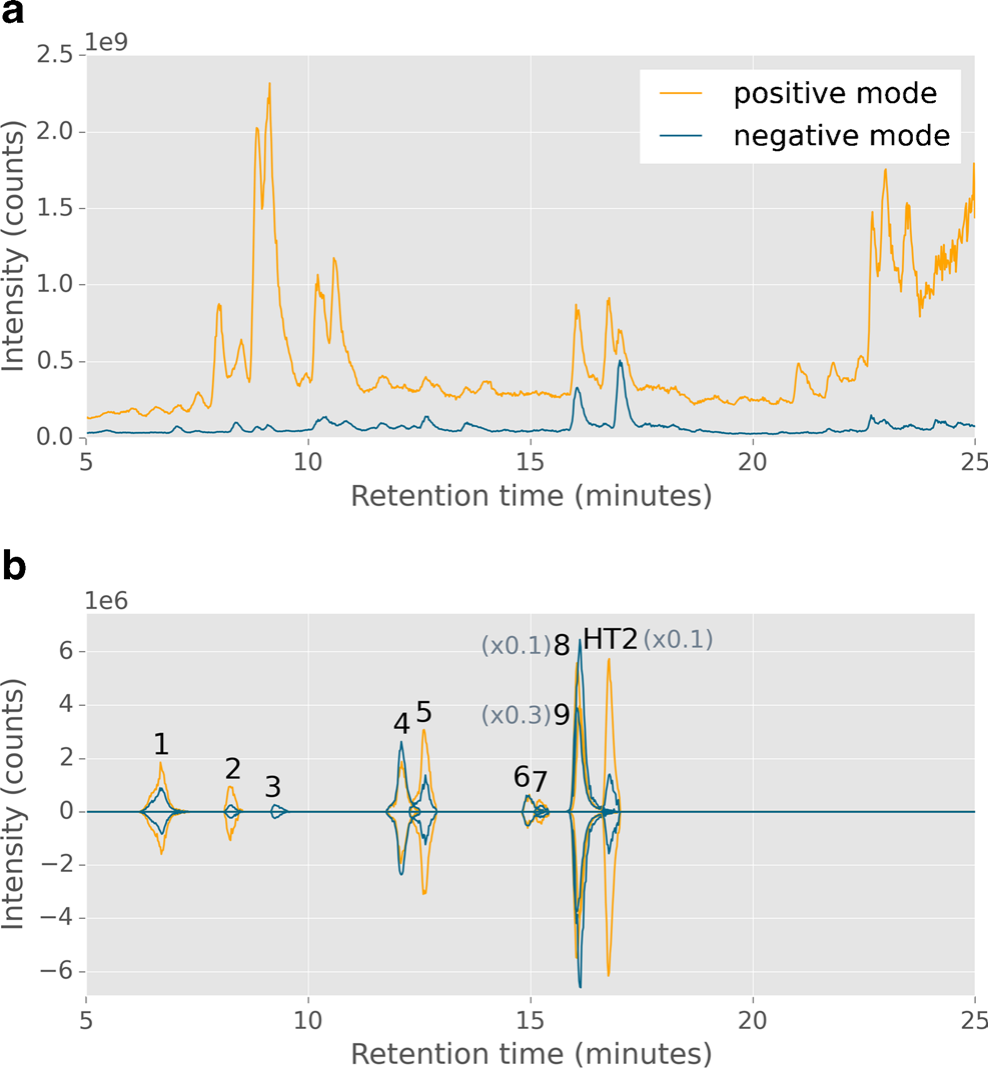
Illustration of fast polarity switching measurement using an Exactive Plus Orbitrap instrument (**a**) and extracted ion chromatograms (*EICs*) based on MetExtract data processing output (**b**). One representative barley sample treated with a 1 + 1 (*v*/*v*) mixture of non-labelled and uniformly ^13^C-labelled HT-2 toxin (5 time points per flowering ear) was used to depict positive (*orange*) and negative (*blue*) total ion chromatogram of Orbitrap measurement and EICs of non-labelled (*up*) and labelled (*down*) HT-2 toxin (*HT2*) as well as its feature groups (*metabolites*) obtained by MetExtract software. *Numbers* above EICs refer to HT-2 toxin metabolites listed in Table 1, and some EICs were scaled down for better visibility of the low abundant metabolites

The total ion chromatogram (TIC) and EICs based on MetExtract data processing output of one representative labelled HT2 barley sample measured with LC-Orbitrap-MS is shown in Fig. 1. After automated data processing by MetExtract, HT2-derived metabolite peaks emerged clearly which are presented in form of EICs.

Application of MetExtract to full scan Orbitrap-derived chromatograms of ^12^C/^13^C-HT2 barley samples revealed features which were grouped according to both retention time and peak shape similarity. Every such feature group represents a distinct metabolite plus unmodified parent toxin HT2. Table 1 summarises all annotated HT2 metabolites in barley. The accurate mass and ion species either of the chosen precursor for the follow-up LC-HRMS/MS experiments or the most abundant ion species for metabolites with low intensity are provided. Due to methodical limitations (e.g. LOD) and the strict criteria of MetExtract software, it is to be expected that especially low abundant metabolites might be missed in some samples. In most cases, this can be explained by the high degree of ^13^C-isotopic enrichment of the parent toxins and thus of its metabolites resulting in missing isotopologues (*M*′ − 1) of the labelled metabolite form. Therefore, even MetExtract hits detected in a single measurement only were considered. On the other hand, MetExtract hits also included false positives whose number increased with higher sample concentration. These false-positive features were successfully excluded from further data evaluation either due to an impossible number of carbon atoms, implausible isotope pattern, imperfect coelution of labelled and corresponding non-labelled compounds or LC-HRMS/MS spectra which were not matching any toxin fragments.

**Table 1:** HT-2 toxin and its (putative) biotransformation products in barley

HT2 metabolites		RT (min)	*m*/*z* ^d^	Ion^d^	Mass accuracy (ppm)	Positive/negative^e^	C-atoms^f^	Sum formula^g^
	HT2^a^	16.8	442.2428	[M + NH_4_]^+^	−1.7	+/−	22	C_22_H_32_O_8_
1	15-Acetyl-T2-tetraol-Glc^b^	6.7	520.2380	[M + NH_4_]^+^	−1.6	+/−	17	C_23_H_34_O_12_
2	15-Acetyl-T2-tetraol-MalGlc^b^	8.3	606.2381	[M + NH_4_]^+^	−1.9	+/−	17	C_26_H_36_O_15_
3	15-Acetyl-T2-tetraol-Glc[–2H]^c^	9.2	545.1867	[M + HCOO]^−^	−1.6	+/−	17	C_23_H_32_O_12_
4	Hydroxy-HT2-Glc^b^	12.1	620.2900	[M + NH_4_]^+^	−2.1	+/−	22	C_28_H_42_O_14_
5	Hydroxy-HT2-MalGlc^b^	12.6	706.2906	[M + NH_4_]^+^	−1.5	+/−	22	C_31_H_44_O_17_
6	T2-triol-Glc^b^	15.0	567.2402	[M + Na]^+^	−1.8	+/−	20	C_26_H_40_O_12_
7a	HT2-di-Glc^b^	15.2	793.3124	[M + HCOO]^−^	−1.5	+/−	22	C_34_H_52_O_18_
7b	HT2-di-Glc^b^	15.8	793.3124	[M + HCOO]^−^	−1.5	+/−	22	C_34_H_52_O_18_
8	HT2-3-*O*-*β*-Glc^a^	16.1	604.2950	[M + NH_4_]^+^	−2.3	+/−	22	C_28_H_42_O_13_
9	HT2-MalGlc^b^	16.1	690.2950	[M + NH_4_]^+^	−2.6	+/−	22	C_31_H_44_O_16_

*RT* retention time, *HT2* HT-2 toxin, *T2* T-2 toxin, *Glc* glucoside, *MalGlc* malonylglucoside

^a^Confirmation with standard by comparison of retention time, accurate mass and HRMS/MS spectra

^b^Annotation with accurate mass, sum formula calculations and HRMS/MS spectra

^c^Annotation with accurate mass and sum formula calculations

^d^Accurate mass and ion species either of the chosen precursor for the follow-up MS/MS experiments or the most abundant ion species for metabolites with low intensity are provided

^e^Detected polarity of compound either by untargeted approach or manually found

^f^Number of C-atoms derived from parent toxin

^g^Sum formula of neutral compound

Measurements in fast polarity switching mode showed that HT2 biotransformation products were found in both applied polarities, and different adducts were formed which facilitated the annotation of the molecular identity of the ion species and increased the probability of MetExtract detection of metabolites with low abundance. Mainly [M + Na]^+^, [M + NH_4_]^+^ and [M + HCOO]^−^ adducts were formed in full scan mass spectra, whilst for the tentative malonylglucoside (MalGlc) derivatives, [M − CO_2_ + HCOO]^−^ adducts dominated in negative mode. Although positive and negative adducts were measured, the HT2 metabolite 15-acetyl-T2-tetraol-Glc[– 2H] was recognised by MetExtract only in negative mode.

### T2 metabolism in barley

MetExtract data evaluation of ^12^C/^13^C-T2 barley samples resulted in features and feature groups. In Table 2, all (putatively) identified T2 derivatives in barley are listed. A comparison with Table 1 depicts that all nine toxin metabolites detected in the HT2-treated samples were also detected in the T2-treated samples, due to fast conversion of T2 into HT2 in planta. For T2, four additional metabolites were detected, namely HT2, T2-Glc, 3-acetyl-T2 and feruloyl-T2. Since the untargeted approach enabled the detection of all HT2 metabolites in T2-treated samples, besides 15-acetyl-T2-tetraol-Glc, 15-acetyl-T2-tetraol-MalGlc and 15-acetyl-T2-tetraol-Glc[– 2H], we manually searched for them by extracting EICs of corresponding ^12^C- and ^13^C-mass signals. This demonstrated that the ^12^C/^13^C signal ratios differed considerably from 1 (up to 6) leading to exclusion by MetExtract. Purity measurements of the T2 test solution showed a contamination with approximately 3 % non-labelled neosolaniol. Thus, the enhanced ^12^C-mass signal might be due to neosolaniol which had been transformed into 15-acetyl-T2-tetraol and its derivatives. From the metabolism study in wheat [13], we got a hint about the presence of one additional T2 metabolite, namely feruloyl-T2. Since it was not recognised by MetExtract because of very low abundance (*M*′ − 1 isotopologue not detected), a manual screening was performed confirming its occurrence.

**Table 2:** T-2 toxin and its (putative) biotransformation products in barley

T2 metabolites		RT (min)	*m*/*z* ^d^	Ion^d^	Mass accuracy (ppm)	Positive/negative^e^	C-atoms^f^	Sum formula^g^
	T2^a^	17.9	484.2532	[M + NH_4_]^+^	−1.9	+	24	C_24_H_34_O_9_
1	15-Acetyl-T2-tetraol-Glc^bh^	6.7	520.2384	[M + NH_4_]^+^	−0.9	+/−	17	C_23_H_34_O_12_
2	15-Acetyl-T2-tetraol-MalGlc^bh^	8.3	606.2382	[M + NH_4_]^+^	−1.7	+/−	17	C_26_H_36_O_15_
3	15-Acetyl-T2-tetraol-Glc[–2H]^ch^	9.3	545.1872	[M + HCOO]^−^	−0.7	+/−	17	C_23_H_32_O_12_
4	Hydroxy-HT2-Glc^b^	12.1	620.2902	[M + NH_4_]^+^	−1.7	+/−	22	C_28_H_42_O_14_
5	Hydroxy-HT2-MalGlc^b^	12.6	706.2912	[M + NH_4_]^+^	−0.7	+/−	22	C_31_H_44_O_17_
6	T2-triol-Glc^b^	14.9	589.2500	[M + HCOO]^−^	−0.3	+/−	20	C_26_H_40_O_12_
7a	HT2-di-Glc^b^	15.2	793.3139	[M + HCOO]^−^	+0.4	+/−	22	C_34_H_52_O_18_
7b	HT2-di-Glc^b^	15.8	793.3139	[M + HCOO]^−^	+0.4	+/−	22	C_34_H_52_O_18_
8	HT2-3-*O*-*β*-Glc^a^	16.1	604.2953	[M + NH_4_]^+^	−1.8	+/−	22	C_28_H_42_O_13_
9	HT2-MalGlc^b^	16.1	690.2952	[M + NH_4_]^+^	−2.3	+/−	22	C_31_H_44_O_16_
10	HT2^a^	16.8	442.2428	[M + NH_4_]^+^	−1.7	+/−	22	C_22_H_32_O_8_
11	T2-Glc^c^	17.0	651.2617	[M + Na]^+^	−1.0	+	24	C_30_H_44_O_14_
12	3-Acetyl-T2^a^	19.1	526.2640	[M + NH_4_]^+^	−1.3	+	24	C_26_H_36_O_10_
13a	Feruloyl-T2^bh^	20.0	665.2557	[M + Na]^+^	−1.7	+	24	C_34_H_42_O_12_
13b	Feruloyl-T2^bh^	20.2	665.2551	[M + Na]^+^	−2.6	+	24	C_34_H_42_O_12_

*RT* retention time, *HT2* HT-2 toxin, *T2* T-2 toxin, *Glc* glucoside, *MalGlc* malonylglucoside

^a^Confirmation with standard by comparison of retention time, accurate mass and HRMS/MS spectra

^b^Annotation with accurate mass, sum formula calculations and HRMS/MS spectra

^c^Annotation with accurate mass and sum formula calculations

^d^Accurate mass and ion species either of the chosen precursor for the follow-up MS/MS experiments or the most abundant ion species for metabolites with low intensity are provided

^e^Detected polarity of compound either by untargeted approach or manually found

^f^Number of C-atoms derived from parent toxin

^g^Sum formula of neutral compound

^h^Not recognised by untargeted approach

Fast polarity switching measurements showed that most T2 biotransformation products were detected in both polarities. On the contrary, under the tested conditions, T2, T2-Glc, 3-acetyl-T2 and feruloyl-T2 were only detected in positive mode as [M + Na]^+^ and [M + NH_4_]^+^ adducts. Moreover, T2-triol-Glc was recognised by MetExtract only in negative ionisation mode.

### Structure annotation of detected HT2 and T2 metabolites

Each metabolite is recorded by MetExtract with the number of C-atoms originating from the studied HT2 or T2. Structure annotation was performed by searching for typical conjugates, by calculating elemental formulas and by involving characteristic fragments detected in HRMS/MS spectra (Table 3). All toxin derivatives, for which LC-HRMS/MS spectra could be obtained in positive mode, showed fragments of HT2 or T2. The fragmentation patterns were very similar to previously reported MS/MS spectra of these toxins [2, 8, 10, 11, 28]. Since mainly Glc and MalGlc derivatives were found, typical fragments of these molecules were used for metabolite characterisation. Mass deviations of precursor ions did not exceed 5 ppm, whilst mass deviations of fragments did not exceed 16 ppm. Further LC-HRMS/MS measurements with pairs of corresponding non-labelled and labelled precursor ions provided additional information for structure annotation. By calculating mass differences between the corresponding fragments, the number of C-atoms remaining from the parent toxin was elucidated. In contrast, according to the native isotopic composition of the plant constituents, there was no ^12^C/^13^C mass shift for fragments derived from glucose, malonylglucose or ferulic acid moieties. As far as standards were available, retention time, accurate mass and MS/MS spectra were compared for identification of the biotransformation products. In the following part, annotation based on LC-HRMS/MS of the individual HT2 and T2 metabolites is described. Primarily, *m*/*z* values experimentally derived from the measurements of the HT2 samples are provided with the exception of those *m*/*z* values, which refer to T2-specific metabolites.

**Table 3:** Characteristic LC-HRMS/MS fragment ions of HT-2 toxin and T-2 toxin, as well as the glucose and malonylglucose moieties used for structure annotation

*m*/*z* ^a^	Ion	Sum formula^b^
HT2 fragments		
323.1489	[HT2 – isoval acid + H]^+^	C_17_H_22_O_6_
263.1278	[HT2 – isoval acid – acetic acid + H]^+^	C_15_H_18_O_4_
245.1172	[HT2 – isoval acid – acetic acid – H_2_O + H]^+^	C_15_H_16_O_3_
233.1172	[C_14_H_16_O_3_ + H]^+^	C_14_H_16_O_3_
215.1067	[HT2 – isoval acid – acetic acid – H_2_O – CH_2_O + H]^+^	C_14_H_14_O_2_
185.0961	[C_13_H_12_O + H]^+^	C_13_H_12_O
T2 fragments		
365.1595	[T2 – isoval acid + H]^+^	C_19_H_24_O_7_
305.1383	[T2 – isoval acid – acetic acid + H]^+^	C_17_H_20_O_5_
245.1172	[T2 – isoval acid – 2 acetic acid + H]^+^	C_15_H_16_O_3_
215.1067	[T2 – isoval acid – 2 acetic acid – CH_2_O + H]^+^	C_14_H_14_O_2_
185.0961	[C_13_H_12_O + H]^+^	C_13_H_12_O
Glucose moiety fragments		
145.0495	[Glucose – 2 H_2_O + H]^+^	C_6_H_8_O_4_
127.0390	[Glucose – 3 H_2_O + H]^+^	C_6_H_6_O_3_
161.0455	[Glucose – H_2_O – H]^−^	C_6_H_10_O_5_
Malonylglucose moiety fragments		
249.0605	[Malonylglucose – H_2_O + H]^+^	C_9_H_12_O_8_
231.0499	[Malonylglucose – 2 H_2_O + H]^+^	C_9_H_10_O_7_
145.0495	[Glucose – 2 H_2_O + H]^+^	C_6_H_8_O_4_
127.0390	[Glucose – 3 H_2_O + H]^+^	C_6_H_6_O_3_
105.0182	[Malonic acid + H]^+^	C_3_H_4_O_4_
161.0455	[Glucose – H_2_O – H]^−^	C_6_H_10_O_5_

*HT2* HT-2 toxin, *T2* T-2 toxin, *isoval acid* isovaleric acid

^a^Exact mass

^b^Sum formula of neutral compound

#### 15-Acetyl-T2-tetraol metabolites (metabolites 1–3)

Our data suggest that barley plants metabolised HT2 or T2 to form putative 15-acetyl-T2-tetraol-Glc (**1**), 15-acetyl-T2-tetraol-MalGlc (**2**) and 15-acetyl-T2-tetraol-Glc[– 2H] (**3**) by the cleavage of an acetyl group at C-4 position in the case of T2, the isovaleryl group at C-8 position and covalent binding of glucose and subsequently malonic acid. MetExtract recognised in full scan mass spectra adducts ([**1** + NH_4_]^+^, *m*/*z* 520.2380; [**2** + NH_4_]^+^, *m*/*z* 606.2381; [**3** + HCOO]^−^, *m*/*z* 545.1867) with a difference of ∆17.057 u (theoretical value) between the non-labelled and labelled metabolite form which corresponds to remaining 17 C-atoms of the HT2 backbone, indicating the loss of the isovaleric acid (isoval acid) moiety minus water (containing five carbon atoms, loss of 84.058 u; theoretical value). LC-HRMS/MS measurements of [M + NH_4_]^+^ and [M + HCOO]^−^ adducts included characteristic fragments of HT2, glucose and malonylglucose moieties as well as fragment *m*/*z* 323.1489 (theoretical value) which corresponds to [15-acetyl-T2-tetraol – H_2_O + H]^+^ (positive LC-HRMS/MS spectra are shown in ESM Figs. S3 and S4). Since intensities for 15-acetyl-T2-tetraol-Glc[– 2H] were too low to obtain meaningful LC-MS/MS spectra, in this case, structure annotation was based on sum formula calculations only. A comparison with 15-acetyl-T2-tetraol-Glc gives a difference of ∆2.014 (theoretical value) equivalent to two H-atoms. It is assumed that after the loss of isovaleric acid minus water, most probably a double bond (i.e. a keto group at C-8 position) was formed.

#### Hydroxy-HT2 metabolites (metabolites 4 and 5)

Tentative hydroxy-HT2 metabolites were most probably formed by hydroxylation of the isovaleryl group and conjugation of a glucose (**4**) and subsequently a malonic acid (**5**) molecule, whilst parent toxin T2 additionally lost the acetyl group at C-4 position. [M + NH_4_]^+^ adducts ([**4** + NH_4_]^+^, *m*/*z* 620.2900; [**5** + NH_4_]^+^, *m*/*z* 706.2906) were measured in full scan mass spectra. The difference of ^12^C and ^13^C metabolite form was ∆22.074 u (theoretical value), indicating an intact HT2 molecule. Positive and negative LC-HRMS/MS measurements of [M + NH_4_]^+^, [M + HCOO]^−^ and [M – H]^−^ ions revealed characteristic fragments of HT2, glucose and malonylglucose moieties (positive LC-HRMS/MS spectra are depicted in ESM Figs. S5 and S6). Furthermore, overlaid MS/MS spectra of corresponding ^12^C and ^13^C-labelled precursor ions showed that *m*/*z* 485.2003 (**4**) and *m*/*z* 571.2020 (**5**) contain 17 C-atoms of HT2 in accordance with [M – O – isoval acid + H]^+^. Additionally, in LC-HRMS/MS spectra of the negative mode (data not shown), a fragment of *m*/*z* 117.0558, corresponding to the [M − H]^−^ ion of isovaleric acid plus one oxygen atom, was observed instead of *m*/*z* 101.0608 ([M − H]^−^ of isovaleric acid; theoretical value) which is typical for HT2 and T2 metabolites containing an unmodified isovaleryl group. The direct measurement of the oxygenated isovaleric acid fragment therefore confirms that the hydroxyl group is located at the isovaleryl group of the detected HT2 derivatives. For the two metabolites of interest, the mass increments between fragment [M – O – isoval acid + H]^+^ and HT2 fragment [HT2 – isoval acid + H]^+^ of ∆162.053 u (**4**) or ∆248.054 u (**5**) correspond to the loss of glucose minus water or the loss of malonylglucose minus water and verify the presence of glucose (**4**) or malonylglucose (**5**), respectively.

#### T2-triol-Glc (metabolite 6)

Barley transformed the parent toxins by cleavage of one acetyl group (C-15 position, in case of HT2) or two acetyl groups (C-4 and C-15 position, in case of T2), respectively. MetExtract software recorded [**6** + Na]^+^ adduct with *m*/*z* 567.2402 and [**6** + HCOO]^−^ adduct with *m*/*z* 589.2500 as well as differences of ∆20.067 u (theoretical value) between the ^12^C and ^13^C peak pairs in LC-HRMS spectra due to a loss of C_2_H_2_O from the parent toxin HT2. The detected fragment [glucose – H_2_O – H]^−^ with *m*/*z* 161.0445 in negative MS/MS spectra of adduct [M + HCOO]^−^ led to the conclusion that tentative T2-triol-Glc was formed.

#### HT2 and T2 glucosides (metabolites 7a, 7b, 8 and 11)

Glucosylation of HT2 and T2 in barley was observed, including identified HT2-3-*O*-*β*-Glc (**8**) as well as putative HT2-di-Glc (**7**) and T2-Glc (**11**) ([**8** + NH_4_]^+^, *m*/*z* 604.2950; [**7** + HCOO]^−^, *m*/*z* 793.3124; [**11** + Na]^+^, *m*/*z* 651.2617). HT2-3-*O*-*β*-Glc and HT2-di-Glc were formed in HT2- as well as in T2-treated (additional loss of acetyl group at C-4 position) ears with a difference of ∆22.074 u (theoretical value) between non-labelled and labelled metabolite forms. LC-HRMS/MS spectra of [M + NH_4_]^+^ (see ESM Figs. S7 and S8) and [M + HCOO]^−^ precursor ions revealed characteristic fragments of HT2 and glucose moieties as well as one or two losses of glucose minus water with ∆162.053 u (theoretical value) calculated from [M + H]^+^ adducts. The observed fragmentation patterns are consistent with the literature [2, 8, 9, 11]. Since the retention time, *m*/*z* of precursor ion and fragmentation pattern of the metabolite HT2-3-*O*-*β*-Glc was compared with those of the HT2-3-*O*-*β*-Glc standard, the structure could be confirmed. The application of another chromatographic method with the longer gradient (gradient method 3) showed that there was only one high metabolite peak which eluted at the same time (15.8 min) as the standard. However, the situation was different for HT2-di-Glc which was annotated by LC-HRMS/MS. Interestingly, two chromatographic peaks of HT2-di-Glc were detected by MetExtract at retention times 15.2 and 15.8 min (gradient method 1; [M + HCOO]^−^, *m*/*z* 793.3124). It is assumed that HT2-di-Glc, as many other metabolites, is derived from HT2-3-*O*-*β*-Glc by the connection of the second glucose molecule via 1,4- or 1,6-glycosidic linkage resulting in two structural isomers. However, without nuclear magnetic resonance measurements, we cannot exclude that the second glucose molecule is located at the available hydroxyl group at the C-4 position of HT2.

T2-Glc was exclusively formed in T2-treated barley. Within positive full scan mass spectra, MetExtract recognised differences of ∆24.081 u (theoretical value) between the ^12^C and ^13^C peak pairs originating from an intact T2 backbone. It was not possible to acquire a meaningful MS/MS spectrum due to the low abundance. A retention time comparison of T2-Glc formed in barley with standard T2-*α*-Glc in triplicate with the long gradient method (gradient method 3) revealed that the structure of metabolite T2-Glc differs from T2-*α*-Glc. Whilst the biotransformation product eluted at 22.2 min, the retention time of the standard was 21.4 min. Our findings contradict those of McCormick et al. [12], who reported the occurrence of T2-*α*-Glc in naturally contaminated oats and wheat. Since the glucoside is most likely formed by a family 1 UDP-glucosyltransferase, which are inverting enzymes, the formation of an alpha glucoside would be very surprising. The difference of retention times indicates that T2 is converted into T2-3-*O*-*β*-Glc in barley under the tested conditions because no other position of T2 is available to bind glucose. The elution order was in accordance with the mentioned study.

#### HT2-MalGlc (metabolite 9)

**Fig. 2:**
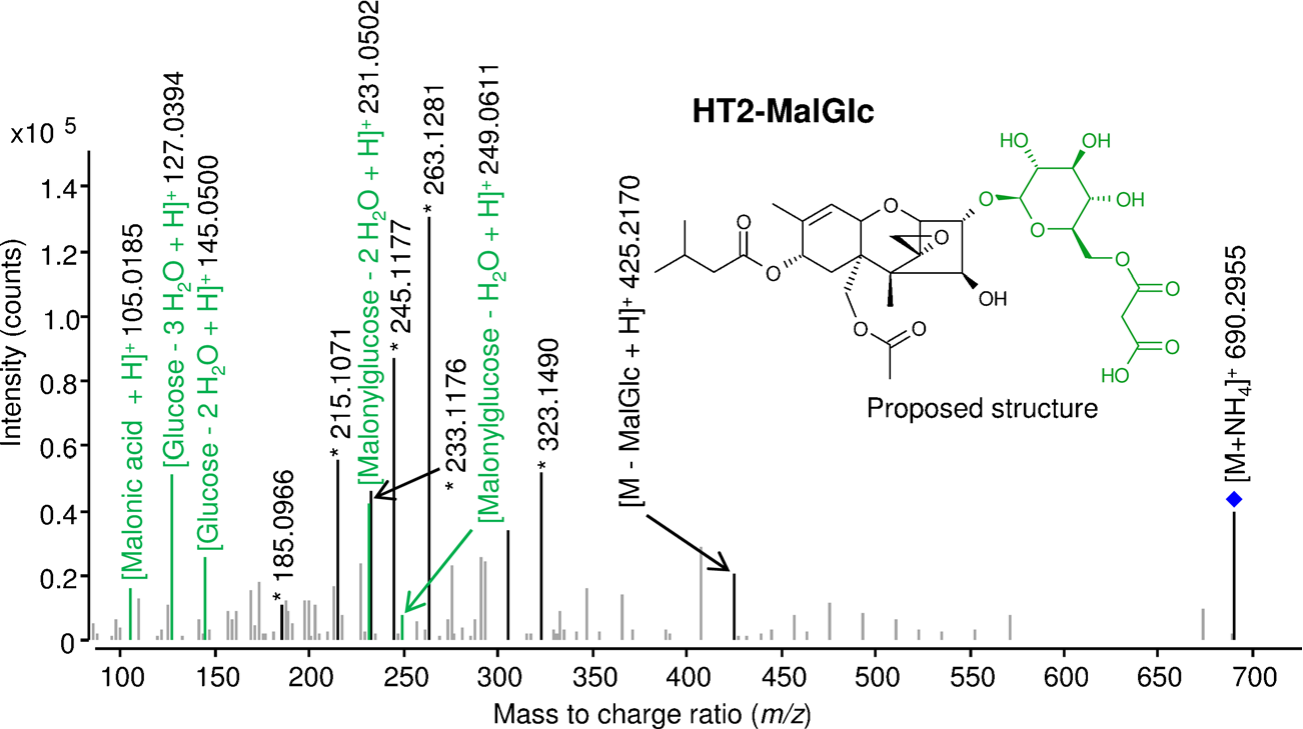
LC-HRMS/MS spectrum of HT-2 toxin-malonylglucoside (HT2-MalGlc), an in planta metabolite of HT-2 toxin and T-2 toxin. Analysis was performed with a 6550 iFunnel Q-TOF LC/MS system in positive electrospray ionisation mode with a collision energy of 16 V. The ammonium adduct was chosen as precursor (marked with a *diamond*). Characteristic fragments used for annotation are *highlighted*, those fragments originating from the conjugate malonylglucose are displayed in *green* and characteristic HT-2 toxin fragments are marked with an *asterisk* (*)

It is suggested that barley plants metabolise HT2 into HT2-3-*O*-*β*-Glc and subsequent into tentative HT2-MalGlc by covalent binding of malonic acid to the glucose moiety. Regarding T2 metabolism, the acetyl group at C-4 position is rapidly cleaved to form HT2 (see below). Within full scan mass spectra, [**9** + NH_4_]^+^ adduct with *m*/*z* 690.2950 was measured. Since the differences of these precursor ions to its ^13^C-mass signals were ∆22.074 u (theoretical value), it was obvious that the parent toxin HT2 was intact. Figure 2 depicts the LC-MS/MS spectrum of HT2-MalGlc in positive mode. Fragmentation of [M + NH_4_]^+^ and [M – H]^−^ adducts show the typical fragments of malonylglucose moiety plus fragment *m*/*z* 425.2170 which corresponds to the putative loss of malonylglucose minus water (∆248.053 u, theoretical value) from the [M + H]^+^ adduct. These results compare favourably with those reported by Kluger et al. [29], who have described a similar fragmentation pattern of deoxynivalenol-MalGlc.

#### HT2 as T2 metabolite (metabolite 10)

T2 was rapidly transformed into HT2 in barley by the loss of an acetyl group at C-4 position. MetExtract detected HT2 with *m*/*z* 442.2428 [**10** + NH_4_]^+^ in full scan mass spectra of T2-treated samples. Since 22 carbon atoms were annotated by MetExtract as well as retention time and LC-MS/MS spectra were in accordance with the authentic standard, HT2 was confirmed as T2 metabolite. LC-HRMS/MS measurement of the [M + NH_4_]^+^ adduct revealed typical fragments of HT2 as listed in Table 3 (LC-MS/MS spectra of HT2 and T2 are shown in ESM Figs. S9 and S10).

#### 3-Acetyl-T2 (metabolite 12)

3-Acetyl-T2 was found only in T2-treated barley and was formed by the conjugation of an acetyl group at the C-3 position. Within positive full scan mass spectra, MetExtract recognised *m*/*z* 526.2640 [**12** + NH_4_]^+^ adduct with a difference of ∆24.081 u (theoretical value) between the non-labelled and labelled metabolite form, indicating that 3-acetyl-T2 is formed from intact T2. Retention time, precursor ion mass and LC-HRMS/MS spectra of the biotransformation product were in accordance with the standard 3-acetyl-T2. The fragmentation of the [M + NH_4_]^+^ adduct mainly generated the loss of isovaleric acid (theoretical value, ∆102.068 u; C_5_H_10_O_2_), acetic acid (theoretical value, ∆60.021 u; C_2_H_4_O_2_) and a ketene (theoretical value, ∆42.011 u; C_2_H_2_O) (positive ion mode LC-HRMS/MS spectrum is shown in ESM Fig. S11).

#### Feruloyl-T2 (metabolites 13a and 13b)

**Fig. 3:**
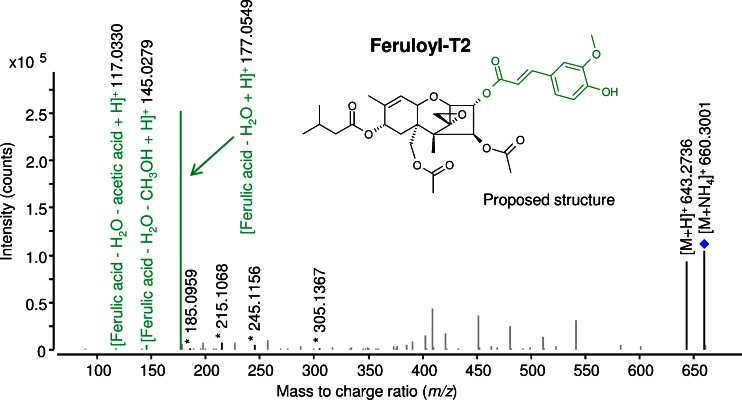
LC-HRMS/MS spectrum of feruloyl-T-2 toxin (feruloyl-T2), an in planta metabolite of T-2 toxin. Analysis was performed with a 6550 iFunnel Q-TOF LC/MS system in positive electrospray ionisation mode with a collision energy of 5 V. The ammonium adduct was chosen as precursor (marked with a *diamond*). Characteristic fragments used for annotation are *highlighted*, those fragments originating from the conjugate ferulic acid are displayed in *green* and characteristic T-2 toxin fragments are marked with an *asterisk* (*)

The presence of this metabolite suggests that ferulic acid was covalently bound to the parent toxin T2. Since we have found feruloyl-T2 initially in T2-treated wheat [13], we manually extracted EICs (*m*/*z* extraction window of ±5 ppm) from Orbitrap raw data of barley as well. Putatively identified feruloyl-T2 is formed from intact T2 in barley because corresponding EICs with a difference of ∆24.081 u ([M + Na]^+^, *m*/*z* 665.2568 and *m*/*z* 689.3373; theoretical values) indicated the intact T2 toxin and showed perfect coelution as well as similar intensities. Two distinct EIC peaks of this metabolite were detected at retention times 20.0 and 20.2 min which might be due to the simultaneous presence of *cis*- and *trans*-ferulic acid conjugates of T2 toxin. Although the *trans*-form of ferulic acid is the main naturally occurring one, the *cis*-form might occur after light-induced non-enzymatic isomerisation [30]. Another isomer option would result from the addition of iso-ferulic acid instead of ferulic acid to T2. Figure 3 shows the fragmentation pattern of this compound. In addition to typical T2 fragments, mass signals of *m*/*z* 177.0549, *m*/*z* 145.0279 and *m*/*z* 117.0330 corresponding to [ferulic acid – H_2_O + H]^+^, [ferulic acid – H_2_O – CH_3_OH + H]^+^ and [ferulic acid – H_2_O – acetic acid + H]^+^ were detected. Since HT2 is a major metabolite of T2, it can be hypothesised that ferulic acid is conjugated to the C-4 position of HT2, whilst the C-3 position is additionally acetylated by the plant or vice versa resulting in compounds with the same molecular weight and putatively similar LC-HRMS/MS spectra. However, with isotopic labelling approach, we can clearly differentiate between groups originating from plant or parent toxin. Since the T2 is fully preserved (∆24.081 u between ^12^C- and ^13^C-mass signals), this hypothesis can be excluded. Thus, T2 appears to be directly and rapidly (see time course experiments) metabolised by the conjugation of ferulic acid at the C-3 position.

#### Screening of isomers of detected HT2 and T2 metabolites

The longer gradient method 3 was developed for LC-Q-TOF-MS analysis to confirm the structures of HT2-3-*O*-*β*-Glc and T2-Glc in comparison with standards. For being able to recognise additional isomers of HT2 and T2 metabolites, which potentially had not been chromatographically separated by LC-Orbitrap-MS analysis, method 3 was applied for the measurement of labelled samples. EICs of corresponding ^12^C/^13^C metabolite ion pairs of all detected biotransformation products were manually extracted, overlaid and checked to confirm coelution, similarity of elution profiles and intensities. As a result, EICs of each of the tentative 15-acetyl-T2-tetraol-MalGlc as well as HT2-MalGlc showed two peaks with one major and one smaller peak (eluting 0.2 or 0.85 min earlier, respectively, approx. 10 % intensity relative to main peak). The observation of these isomers may result from the conjugation of malonic acid to different hydroxyl groups of glucose. Interestingly, tentative hydroxy-HT2-Glc and hydroxy-HT2-MalGlc which are presumably derived from HT2-3-*O*-*β*-Glc were detected in barley in form of three isomers. One major peak and two smaller peaks (eluting 0.15 and 0.25 min earlier, respectively, approx. 10–20 % intensity relative to major peak) were revealed, suggesting that the additional hydroxyl group is located at different positions of the HT2 backbone or, in case of MalGlc derivative, malonic acid is conjugated to different hydroxyl groups of glucose.

### Method validation for quantification

*R*_A_, SSE and *R*_E_ were determined for HT2, T2, HT2-3-*O*-*β*-Glc, 3-acetyl-T2 and T2-Glc. *R*_A_ was very similar to SSE in undiluted matrix solution. Therefore, calculated *R*_E_ of the five quantified compounds was between 94 and 109 % with RSDs ≤ 9 % (*n* = 3). Matrix effects for 1 + 9 (*v*/*v*) and 1 + 49 (*v*/*v*) dilutions of the extraction solution were in the range of 98–114 % (RSDs ≤ 4 %, *n* = 3) and consequently negligible for the intended purpose to estimate the toxin metabolism rates. However, for undiluted samples, SSE values between 88 and 144 % (RSD ≤ 5 %, *n* = 3) were observed due to matrix components which affect the ionisation process of the coeluting metabolites of interest (see ESM Table S12).

### Time courses and mass balances of HT2, T2 and its metabolites

**Fig. 4:**
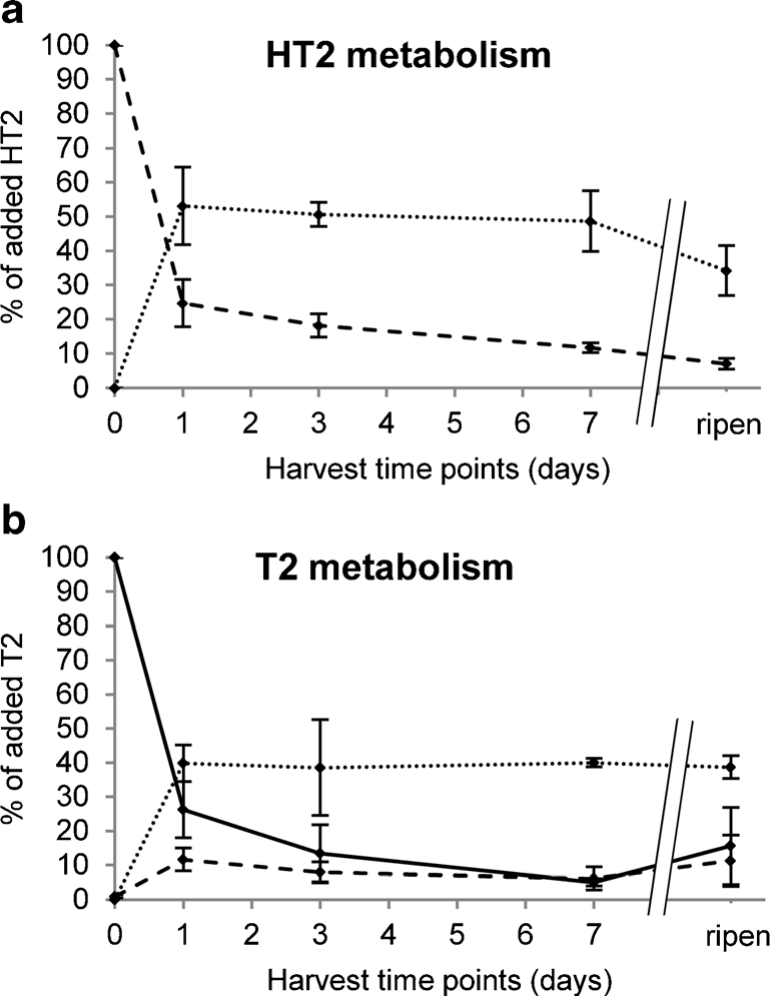
Time courses of quantified HT-2 toxin (HT2, *broken line*), T-2 toxin (T2, *continuous line*) and HT-2 toxin-3-*O*-*β*-glucoside (HT2-3-*O*-*β*-Glc, *dotted line*) depicted separately for HT2- (**a**) and T2-treated (**b**) barley ears. Ears were treated with 200 μg toxin and harvested immediately, 1, 3 and 7 days after treatment and at full-ripening stage. For each ear, absolute analyte concentrations were measured and related to the amount of theoretically added toxin and plotted versus harvest time point after treatment. Analysis was performed with a 6550 iFunnel Q-TOF LC/MS system. *Error bars* refer to the standard deviation of biological triplicates

Time courses of quantified HT2, T2 and HT2-3-*O*-*β*-Glc are shown in Fig. 4. It was observed that the recovery of added HT2 and T2 at time point 0 (harvest and quenching immediately after treatment) deviated significantly from the expected 100 %. We presume that the time period allowed for the toxin to diffuse into the plant cells was too short, and thus, sample handling for harvest and quenching resulted in a loss of toxin solution (possible wash off of toxin by liquid nitrogen and the contact with gloves and scissors also contributed to losses). Standard deviations for time point 0 (*n* = 3) were considerably higher than those of any other time point which supports this assumption. Therefore, the theoretically added toxin amount of 200 μg HT2 (equal to 0.471 μmol) and T2 (0.429 μmol) at time point 0 was used as starting point to calculate percent yield of the respective derivatives formed at later time points. Figure 4 shows that HT2-3-*O*-*β*-Glc has been found to be the main metabolite of both HT2 and T2 toxin which reached its maximum already 1 day after toxin treatment.

#### Kinetics of HT2 metabolism

**Fig. 5:**
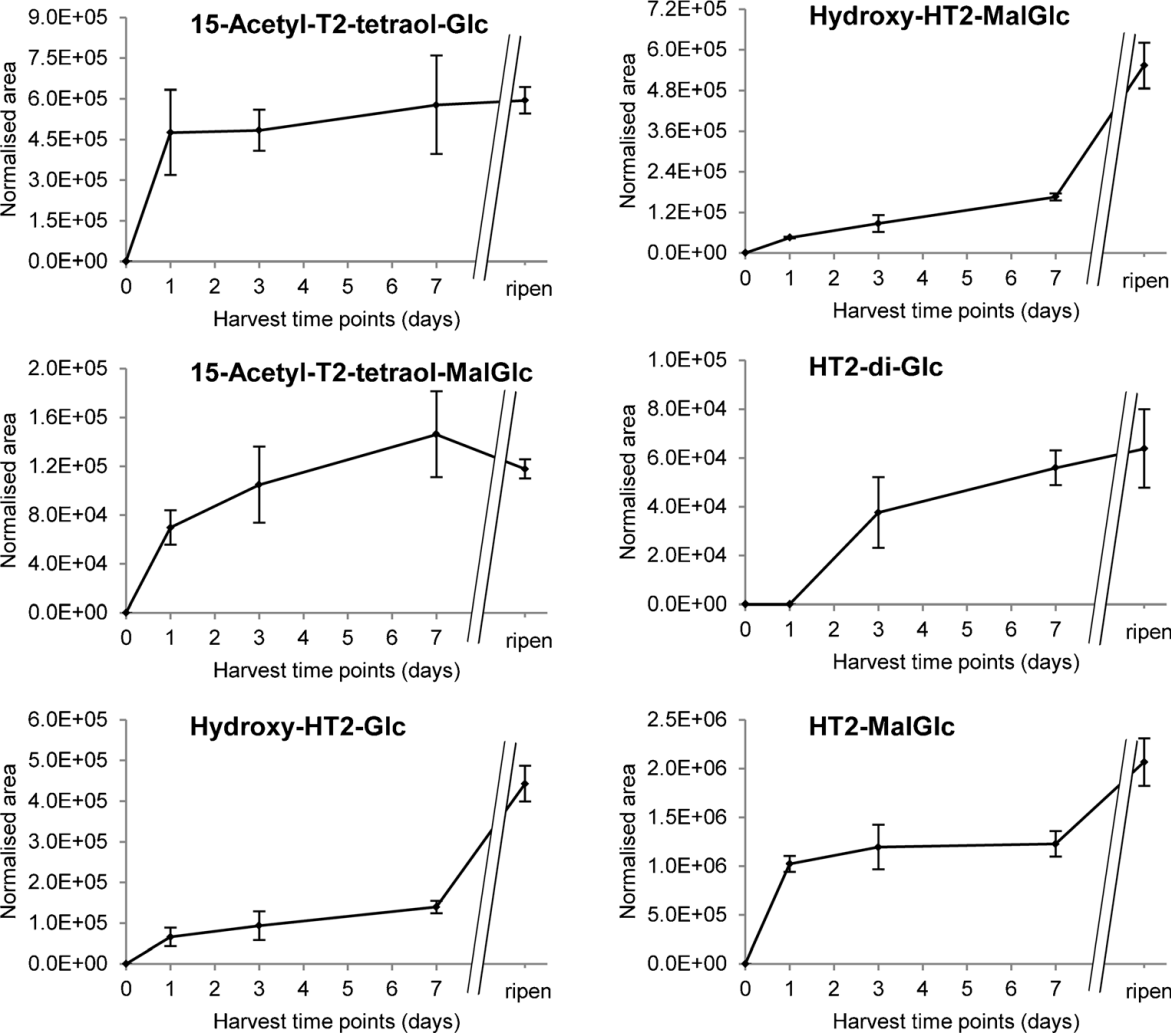
Relative time courses of HT-2 toxin-derived metabolites. Barley ears were treated with 200 μg HT-2 toxin and harvested immediately, 1, 3 and 7 days after treatment and at full-ripening stage. Relative amounts (areas of extracted ion chromatogram peaks normalised by ear weight) are plotted versus harvest time point after treatment. Analysis was performed with a 6550 iFunnel Q-TOF LC/MS system. *Error bars* refer to the standard deviation of biological triplicates. *HT2* HT-2 toxin, *T2* T-2 toxin, *Glc* glucoside, *MalGlc* malonylglucoside

Regarding HT2, approximately 53 % (0.250 ± 0.054 μmol) was transformed to HT2-3-*O*-*β*-Glc, whilst 25 % (0.116 ± 0.033 μmol) remained unmodified as parent toxin within the first 24 h upon treatment. With increasing time, a decrease of HT2 and HT2-3-*O*-*β*-Glc was observed which ended in a content of 7 % (0.033 ± 0.007 μmol) and 34 % (0.161 ± 0.034 μmol) relative to the originally added HT2 after ripening, respectively. This finding confirms that HT2-3-*O*-*β*-Glc is further metabolised and correlates well with the relative quantification (Fig. 5) of HT2 metabolites. Taking a closer look at the formation of HT2 biotransformation products over time, hydroxy-HT2-Glc, hydroxy-HT2-MalGlc, HT2-di-Glc, HT2-MalGlc, as well as T2-triol-Glc (detectable only at one time point) show maximal abundance after ripening, leading to the assumption that they are derived from early formed HT2-3-*O*-*β*-Glc. Moreover, 15-acetyl-T2-tetraol-Glc and 15-acetyl-T2-tetraol-MalGlc were also found to be produced after 1 day. Although absolute quantification was not possible for HT2-MalGlc due to the lack of an authentic standard, comparison of EIC peak areas suggests that it belongs to the major biotransformation products.

#### Kinetics of T2 metabolism

**Fig. 6:**
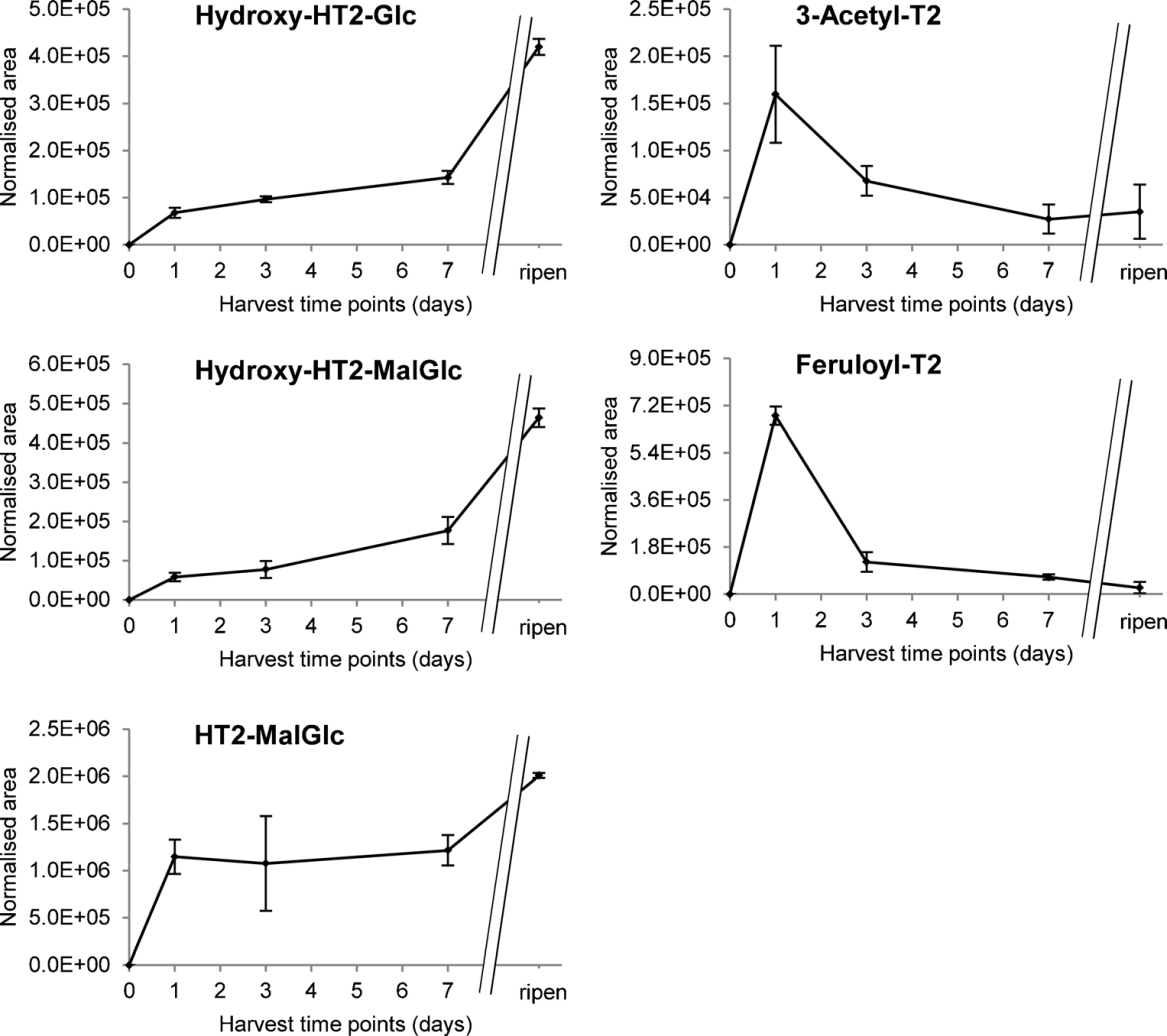
Relative time courses of T-2 toxin-derived metabolites. Barley ears were treated with 200 μg T-2 toxin and harvested immediately, 1, 3 and 7 days after treatment and at full-ripening stage. Relative amounts (areas of extracted ion chromatogram peaks normalised by ear weight) are plotted versus harvest time point after treatment. Analysis was performed with a 6550 iFunnel Q-TOF LC/MS system. *Error bars* refer to the standard deviation of biological triplicates. *HT2* HT-2 toxin, *T2* T-2 toxin, *Glc* glucoside, *MalGlc* malonylglucoside

One day after treatment of barley plants with 200 μg T2 (0.429 μmol), approximately 26 % of unmodified T2 (0.113 ± 0.035 μmol) were still present, whilst 12 % (0.050 ± 0.014 μmol) had been converted into HT2 and 40 % (0.171 ± 0.023 μmol) into HT2-3-*O*-*β*-Glc. As the sum of these three compounds amounts to 78 % of the initially added T2, all other biotransformation products of T2 made up a maximum of 22 % at this time point. 3-Acetyl-T2 was quantified with approximately 0.058 % (0.249 ± 0.073 nmol) of the originally added T2, whilst T2-Glc reached a maximum of approximately 0.065 % (0.278 nmol; only one sample at time point 1 day showed levels > LOQ). Quantification of samples which had been harvested 3 and 7 days after treatment showed that the amount of T2 and HT2 had been further decreased, whilst the quickly formed HT2-3-*O*-*β*-Glc remained almost constant. It is assumed that T2 is very rapidly transformed into HT2 in planta and subsequently converted into HT2-3-*O*-*β*-Glc being the derivative which is then further metabolised. Time courses of relative abundance of T2 metabolites are shown in Fig. 6. Hydroxy-HT2-Glc, hydroxy-HT2-MalGlc, T2-triol-Glc (detectable only at one time point), HT2-di-Glc (detectable only at one time point) and HT2-MalGlc showed the highest amounts after ripening, suggesting that they are formed from HT2-3-*O*-*β*-Glc. Interestingly however, the conjugates carrying intact T2 toxin, namely T2-Glc (detectable only at one time point), 3-acetyl-T2 and feruloyl-T2, all attained their maximum levels after 1 day of incubation. This observation clearly supports the assumption that conjugation of T2 occurs immediately after application of T2 toxin, before the parent toxin is hydrolysed to HT2.

### Detoxification of HT2 and T2

It was observed that barley modifies HT2 and T2 by using phase I as well as phase II metabolism processes. On the one hand, hydrolysis of the non-polar acetyl and isovaleryl groups and on the other hand hydroxylation and covalent binding of glucose and malonic acid occurred. Therefore, it is apparent that as part of the detoxification process, the HT2- and T2-treated plants try to inactivate these xenobiotics by transforming them into more polar compounds. Masuda et al. [31] reported that phytotoxicity of HT2 and T2 is comparable, whilst similar compounds without an isovaleryl group at the C-8 position induce only minor phytotoxic effects. Since glucosylation of the type B trichothecene deoxynivalenol was confirmed to be an important detoxification mechanism of plants [29], it is suggested that the glucosylated forms of HT2 and T2 are also less phytotoxic than the parent toxins. In contrast to deoxynivalenol plant metabolism, no glutathione conjugation was observed. A study [32] showing that trichothecenes with a hydroxyl group at C-3 position are more phytotoxic than those with an acetyl group indicates that acetylation of T2 and probably conjugation of ferulic acid at C-3 position provide an additional way for detoxification. This is also in good agreement with the observation that the metabolic modification at the C-3 of HT2 and T2 is potentially dominating (shown for conjugation of glucose to HT2). Further biological interpretation of HT2 and T2 plant metabolism is included in the study performed with wheat [13].

## Conclusion

In this study, an analytical strategy based on isotopic labelling, LC-Orbitrap-MS analysis in fast polarity switching mode and data processing by MetExtract software was used for elucidation of HT2 and T2 metabolism in barley. Measurements in both electrospray polarities yielded complementary information which was shown to be highly valuable with respect to both coverage as well as annotation of metabolites. The qualitative data evaluation strategy with stable isotopic labelling and the application of MetExtract software enabled the untargeted analysis by exclusive extraction of HT2- and T2-derived biotransformation products and supported their structure annotation by supplying ^12^C/^13^C mass shifts in MS as well as MS/MS spectra. MetExtract provides an easy, rapid, sensitive and specific detection method. Since the Exactive Plus Orbitrap was not equipped with a collision cell, further MS/MS-based structure annotation experiments were performed with a LC-Q-TOF instrument. Therefore, it was possible to annotate and partly identify 9 HT2 and 13 T2 metabolites. The metabolism routes included hydrolysis of acetyl and isovaleryl groups, hydroxylation as well as covalent binding of glucose, malonic acid, acetic acid and ferulic acid. Additionally, putative isomers of 15-acetyl-T2-tetraol-MalGlc, hydroxy-HT2-Glc, hydroxy-HT2-MalGlc, HT2-di-Glc, HT2-MalGlc and feruloyl-T2 (two or three isomers for each) were revealed. As a result of the time course experiments, HT2-3-*O*-*β*-Glc was verified as the major metabolite of HT2 and T2 metabolism which reached its maximum already 1 day after toxin treatment and was subsequently further metabolised.

## Electronic supplementary material

ESM 1 (PDF 200 kb)
