# Supplementary material for: Tracing the metabolism of HT-2 toxin and T-2 toxin in barley by isotope-assisted untargeted screening and quantitative LC-HRMS analysis
Source: Anal Bioanal Chem. 2015 Sep 3;407(26):8019–33. doi: 10.1007/s00216-015-8975-9 (PMC4595538; doi:10.1007/s00216-015-8975-9)
Supplement: Supplementary file 1 — (PDF 200 kb) [file 216_2015_8975_MOESM1_ESM.pdf]

Analytical and Bioanalytical Chemistry

Electronic Supplementary Material

Tracing the metabolism of HT-2 toxin and T-2 toxin in barley by isotope assisted untargeted screening and quantitative LC-HRMS analysis

Jacqueline Meng-Reiterer, Elisabeth Varga, Alexis V. Nathanail, Christoph Bueschl, Justyna Rechthaler, Susan P. McCormick, Herbert Michlmayr, Alexandra Malachová, Philipp Fruhmann, Gerhard Adam, Franz Berthiller, Marc Lemmens, Rainer Schuhmacher

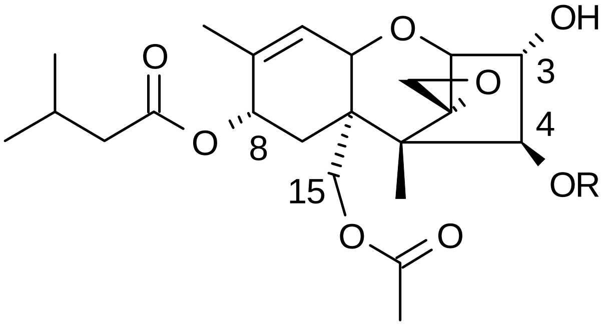

Fig. S1 Structural formula of HT-2 toxin (R = H) and T-2 toxin (R = acetyl). Most relevant positions for this plant metabolism study are highlighted.

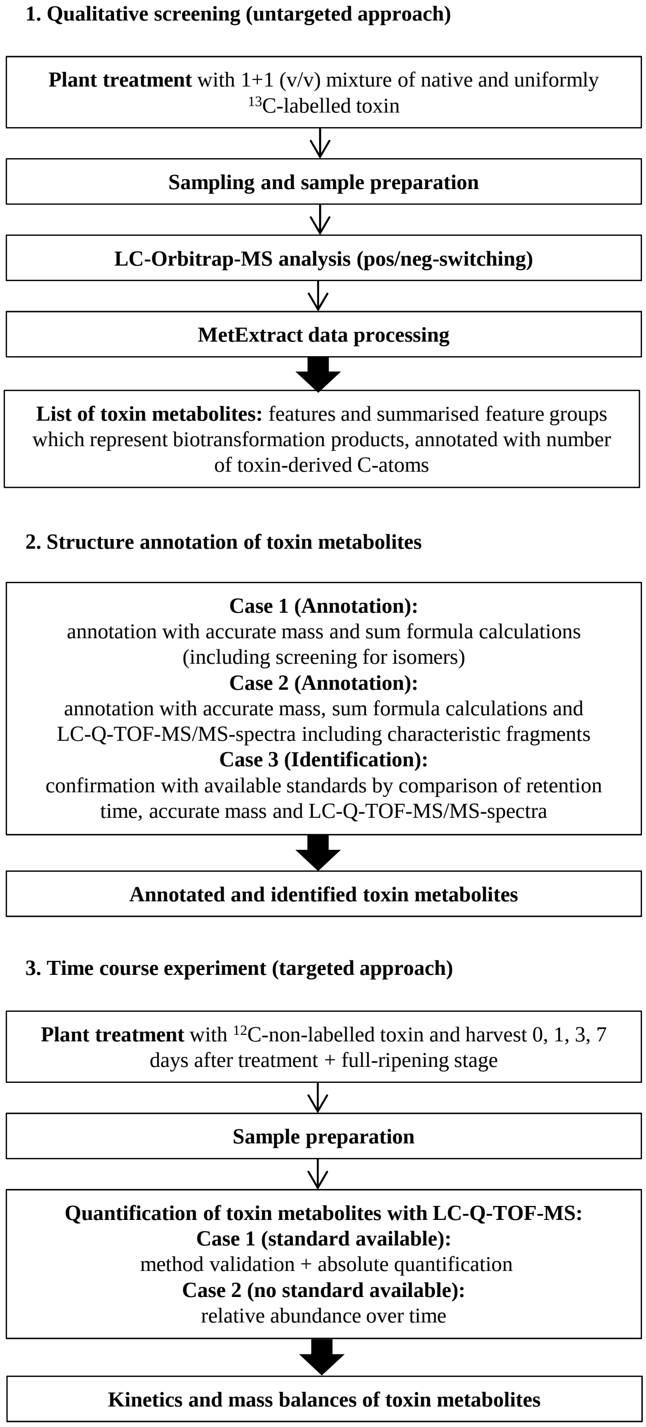

Fig. S2 Workflow for the study of the metabolism of HT-2 toxin and T-2 toxin in barley. Liquid chromatography coupled to high resolution mass spectrometry (LC-HRMS(/MS)) was used for qualitative screening, structure annotation and (relative) quantification.

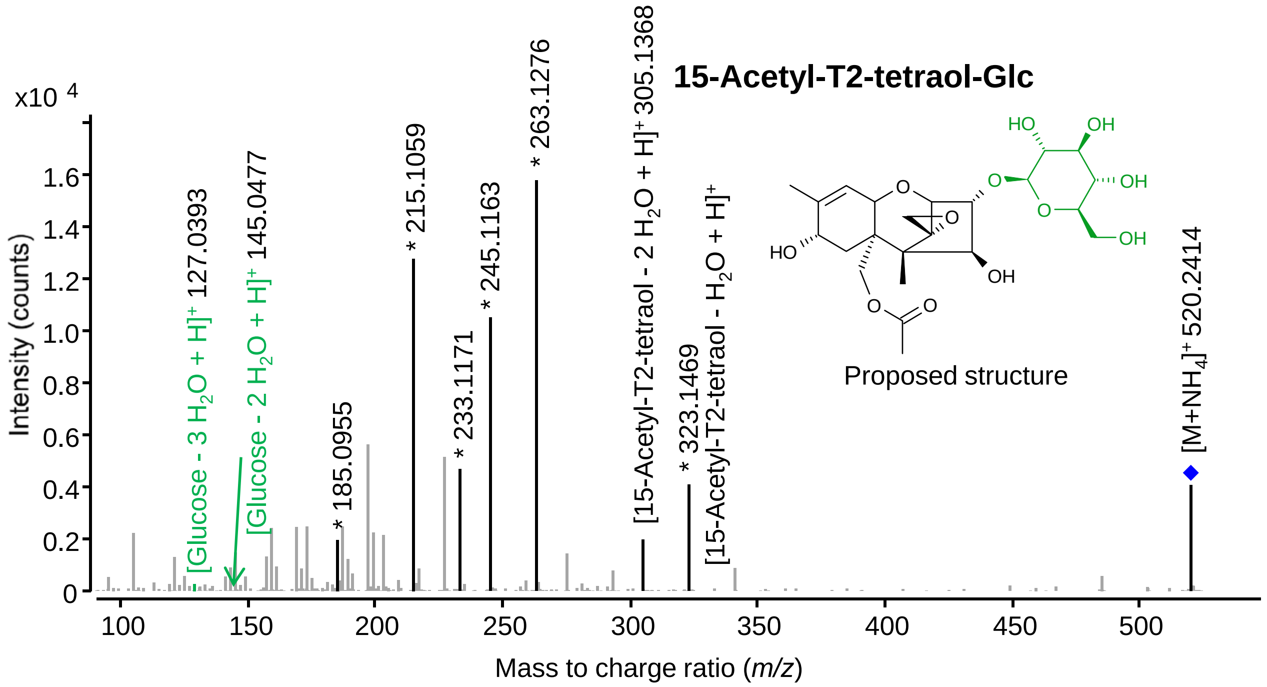

Fig. S3 LC-HRMS/MS-spectrum of 15-acetyl-T2-tetraol-Glc, an *in planta* metabolite of HT-2 toxin and T-2 toxin. Analysis was performed with a 6550 iFunnel Q-TOF LC/MS system in positive electrospray ionisation mode with a collision energy of 10 V. The ammonium adduct was chosen as precursor (marked with a diamond). Characteristic fragments used for annotation are highlighted, those fragments originating from the conjugate glucose are displayed in green and characteristic HT-2 toxin fragments are marked with an asterisk (*). T2 (T-2 toxin), Glc (glucoside)

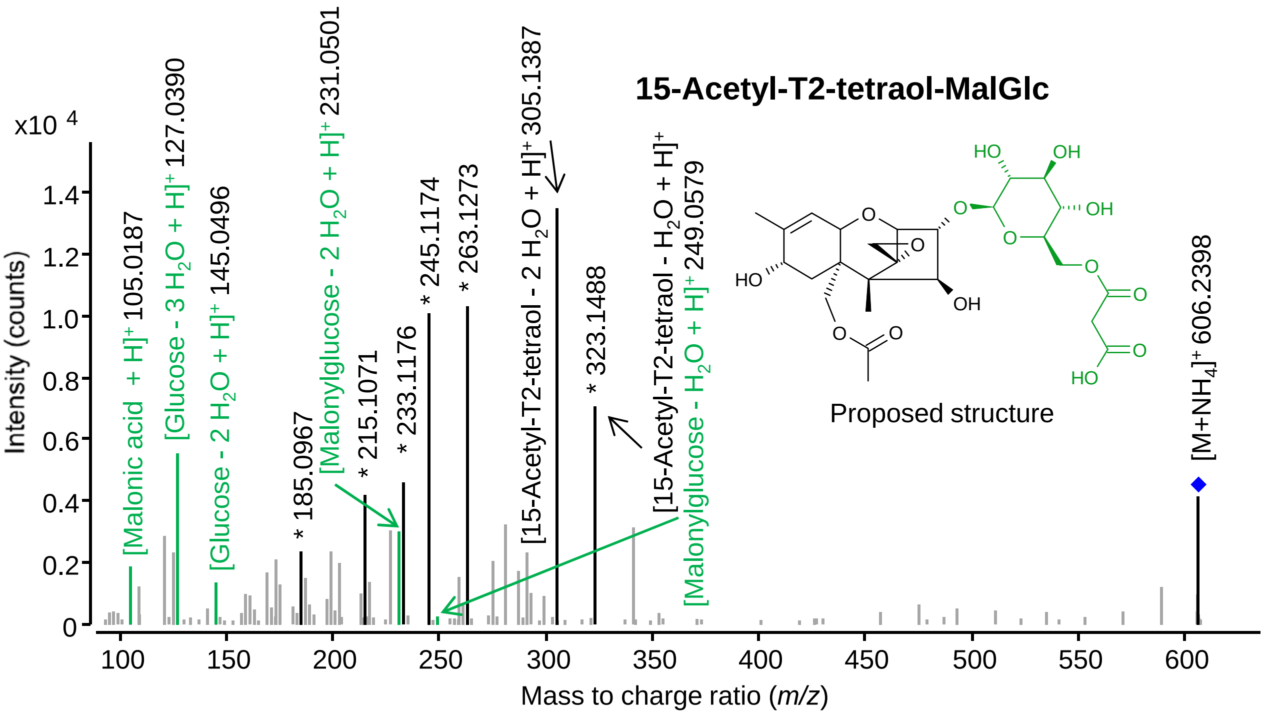

Fig. S4 LC-HRMS/MS-spectrum of 15-acetyl-T2-tetraol-MalGlc, an *in planta* metabolite of HT-2 toxin and T-2 toxin. Analysis was performed with a 6550 iFunnel Q-TOF LC/MS system in positive electrospray ionisation mode with a collision energy of 16 V. The ammonium adduct was chosen as precursor (marked with a diamond). Characteristic fragments used for annotation are highlighted, those fragments originating from the conjugate malonylglucose are displayed in green and characteristic HT-2 toxin fragments are marked with an asterisk (*). T2 (T-2 toxin), MalGlc (malonylglucoside)

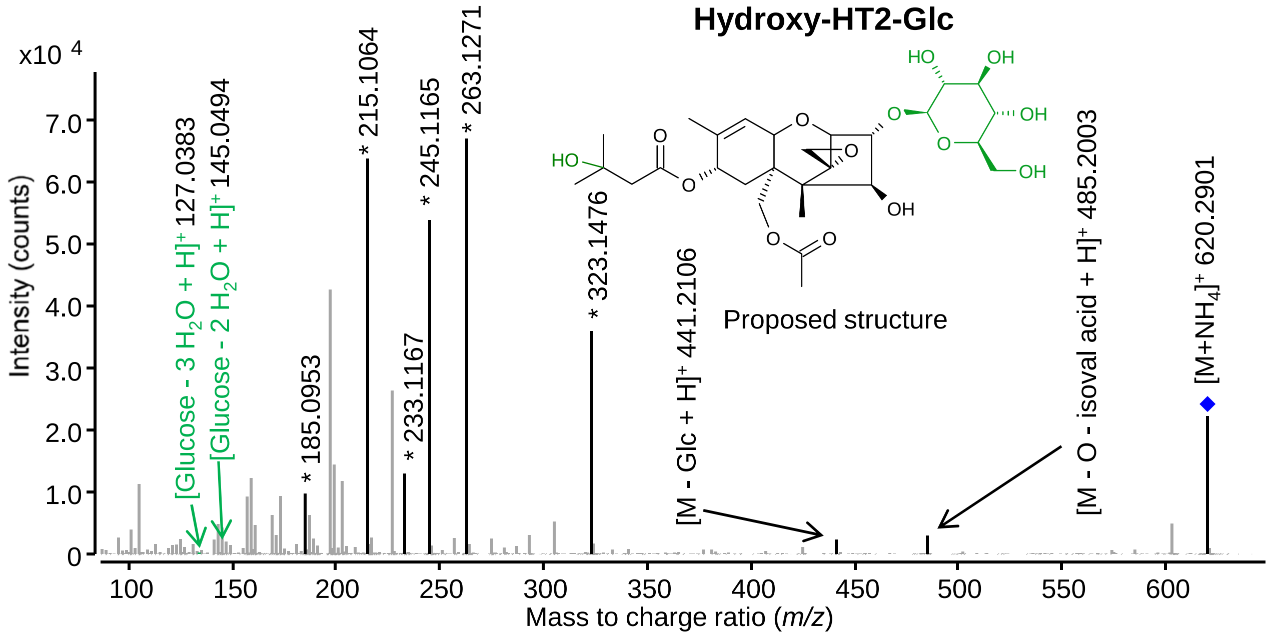

Fig. S5 LC-HRMS/MS-spectrum of hydroxy-HT2-Glc, an *in planta* metabolite of HT-2 toxin and T-2 toxin. Analysis was performed with a 6550 iFunnel Q-TOF LC/MS system in positive electrospray ionisation mode with a collision energy of 10 V. The ammonium adduct was chosen as precursor (marked with a diamond). Characteristic fragments used for annotation are highlighted, those fragments originating from the conjugate glucose are displayed in green and characteristic HT-2 toxin fragments are marked with an asterisk (*). HT2 (HT-2 toxin), Glc (glucoside), isoval acid (isovaleric acid)

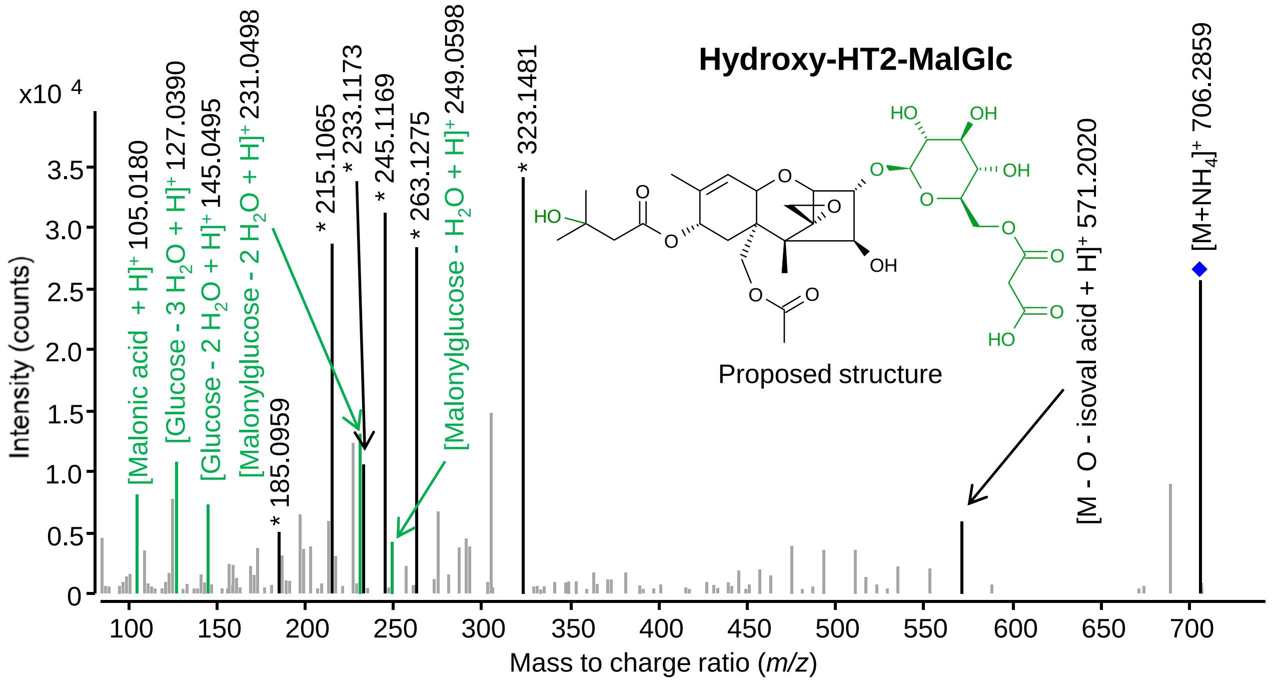

Fig. S6 LC-HRMS/MS-spectrum of hydroxy-HT2-MalGlc, an *in planta* metabolite of HT-2 toxin and T-2 toxin. Analysis was performed with a 6550 iFunnel Q-TOF LC/MS system in positive electrospray ionisation mode with a collision energy of 16 V. The ammonium adduct was chosen as precursor (marked with a diamond). Characteristic fragments used for annotation are highlighted, those fragments originating from the conjugate malonylglucose are displayed in green and characteristic HT-2 toxin fragments are marked with an asterisk (*). HT2 (HT-2 toxin), MalGlc (malonylglucoside), isoval acid (isovaleric acid)

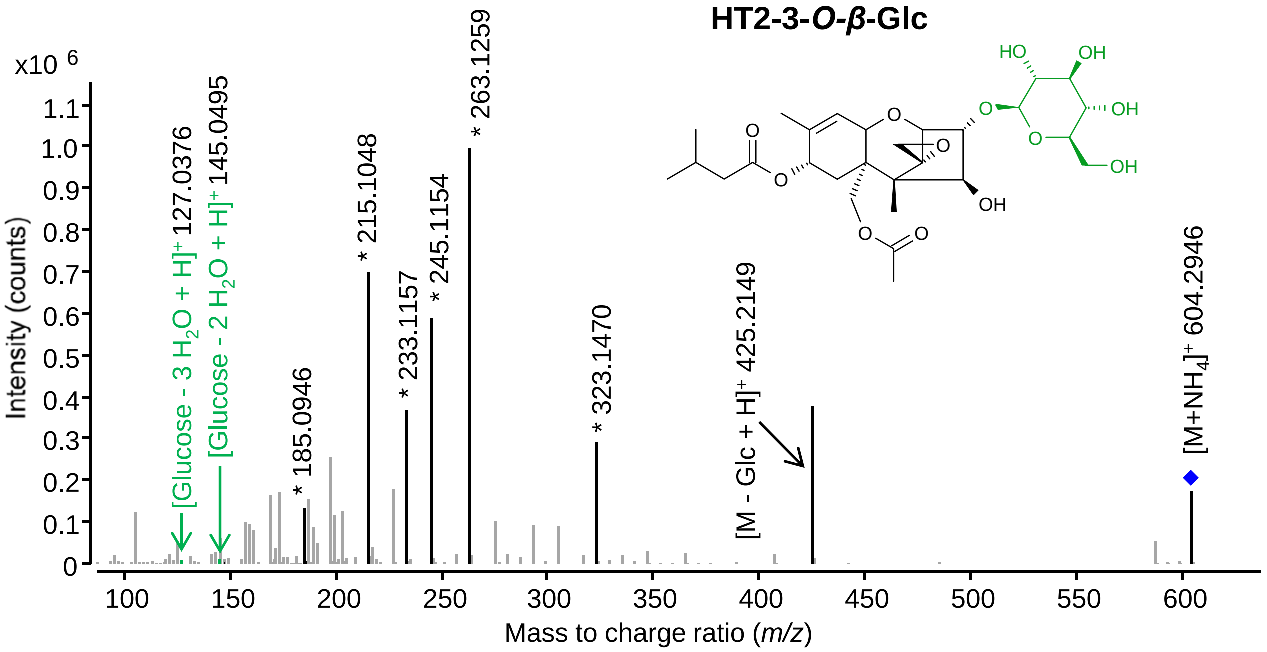

Fig. S7 LC-HRMS/MS-spectrum of HT2-3-*O*-*β*-Glc, an *in planta* metabolite of HT-2 toxin and T-2 toxin. Analysis was performed with a 6550 iFunnel Q-TOF LC/MS system in positive electrospray ionisation mode with a collision energy of 10 V. The ammonium adduct was chosen as precursor (marked with a diamond). Characteristic fragments used for annotation are highlighted, those fragments originating from the conjugate glucose are displayed in green and characteristic HT-2 toxin fragments are marked with an asterisk (*). HT2 (HT-2 toxin), Glc (glucoside)

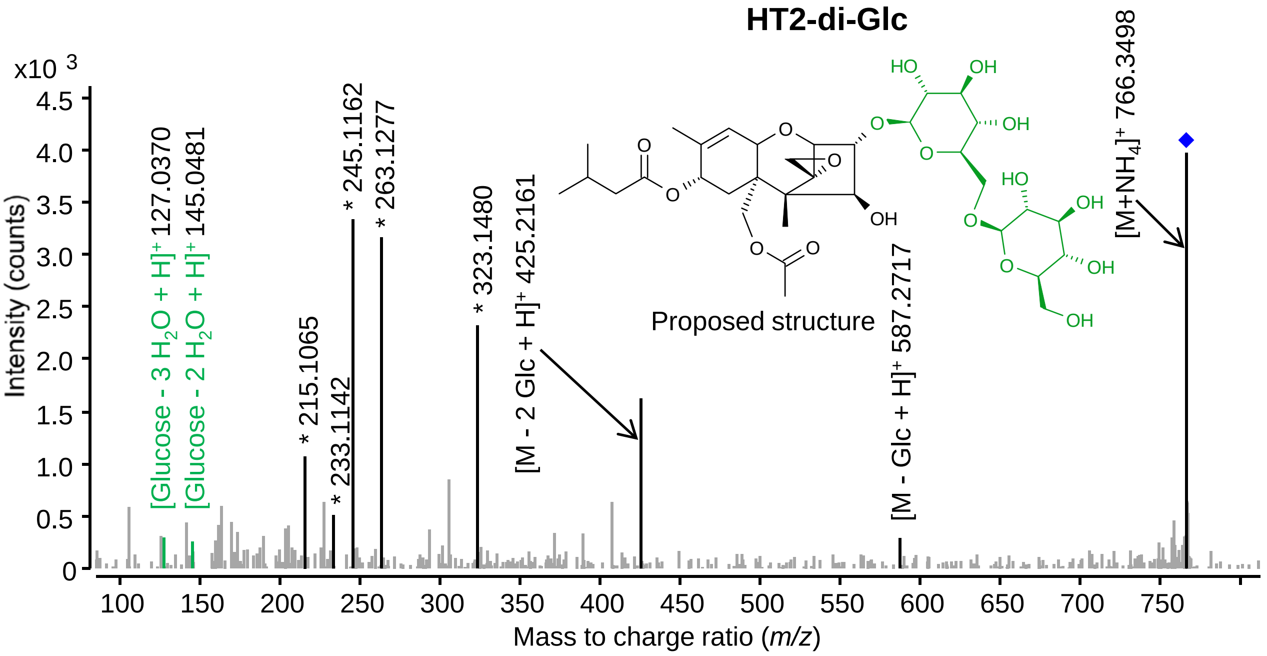

Fig. S8 LC-HRMS/MS-spectrum of HT2-di-Glc, an *in planta* metabolite of HT-2 toxin and T-2 toxin. Analysis was performed with a 6550 iFunnel Q-TOF LC/MS system in positive electrospray ionisation mode with a collision energy of 10 V. The ammonium adduct was chosen as precursor (marked with a diamond). Characteristic fragments used for annotation are highlighted, those fragments originating from the conjugate glucose are displayed in green and characteristic HT-2 toxin fragments are marked with an asterisk (*). HT2 (HT-2 toxin), Glc (glucoside)

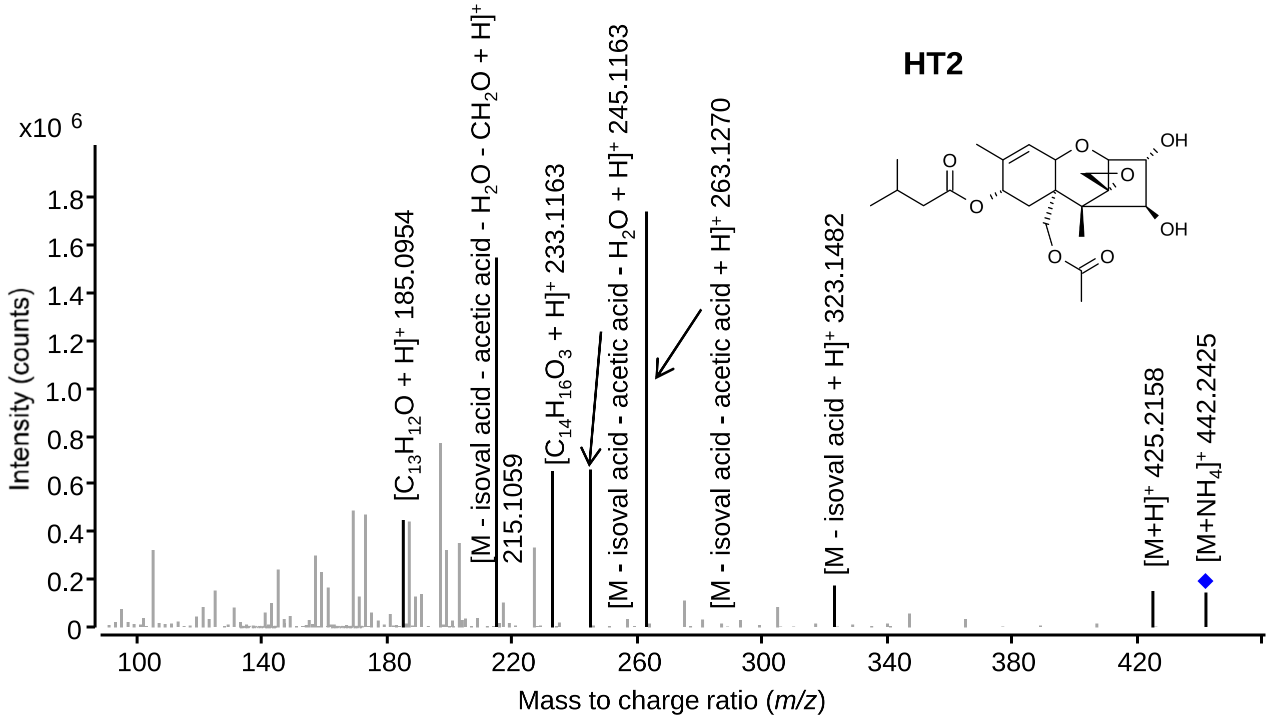

Fig. S9 LC-HRMS/MS-spectrum of HT-2 toxin, an *in planta* metabolite of T-2 toxin. Analysis was performed with a 6550 iFunnel Q-TOF LC/MS system in positive electrospray ionisation mode with a collision energy of 10 V. The ammonium adduct was chosen as precursor (marked with a diamond). Characteristic fragments used for annotation are highlighted. HT2 (HT-2 toxin), isoval acid (isovaleric acid)

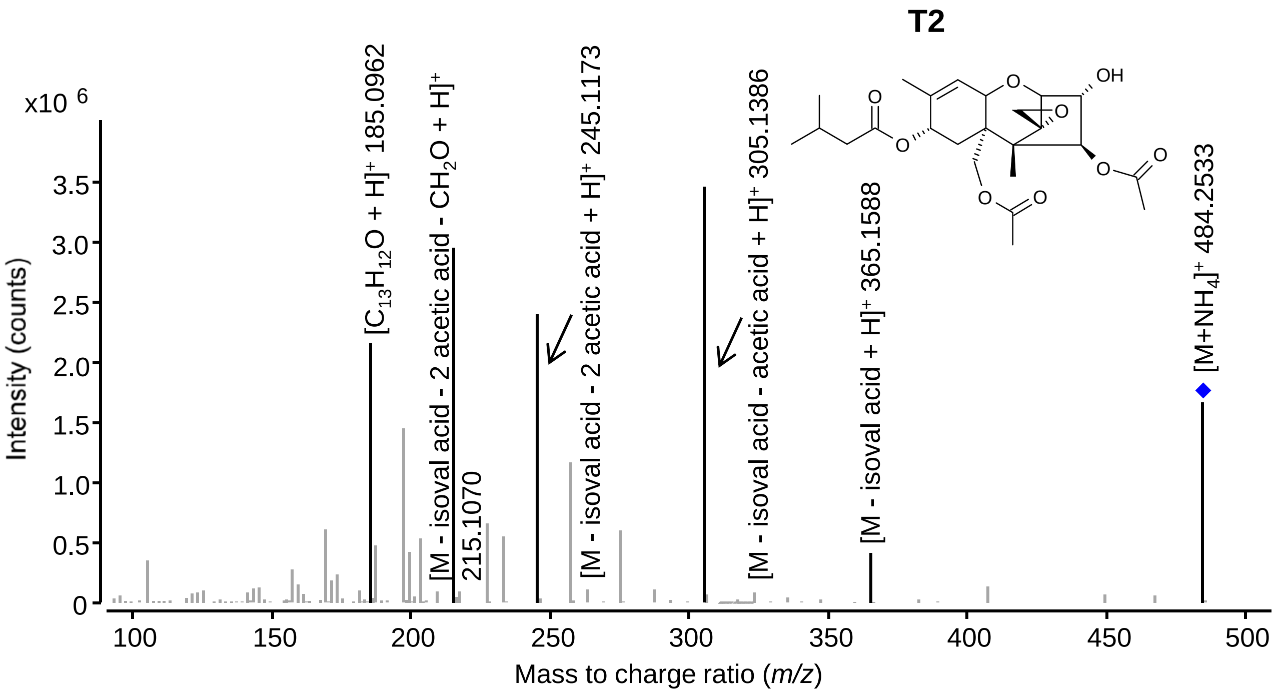

Fig. S10 LC-HRMS/MS-spectrum of T-2 toxin. Analysis was performed with a 6550 iFunnel Q-TOF LC/MS system in positive electrospray ionisation mode with a collision energy of 10 V. The ammonium adduct was chosen as precursor (marked with a diamond). Characteristic fragments used for annotation are highlighted. T2 (T-2 toxin), isoval acid (isovaleric acid)

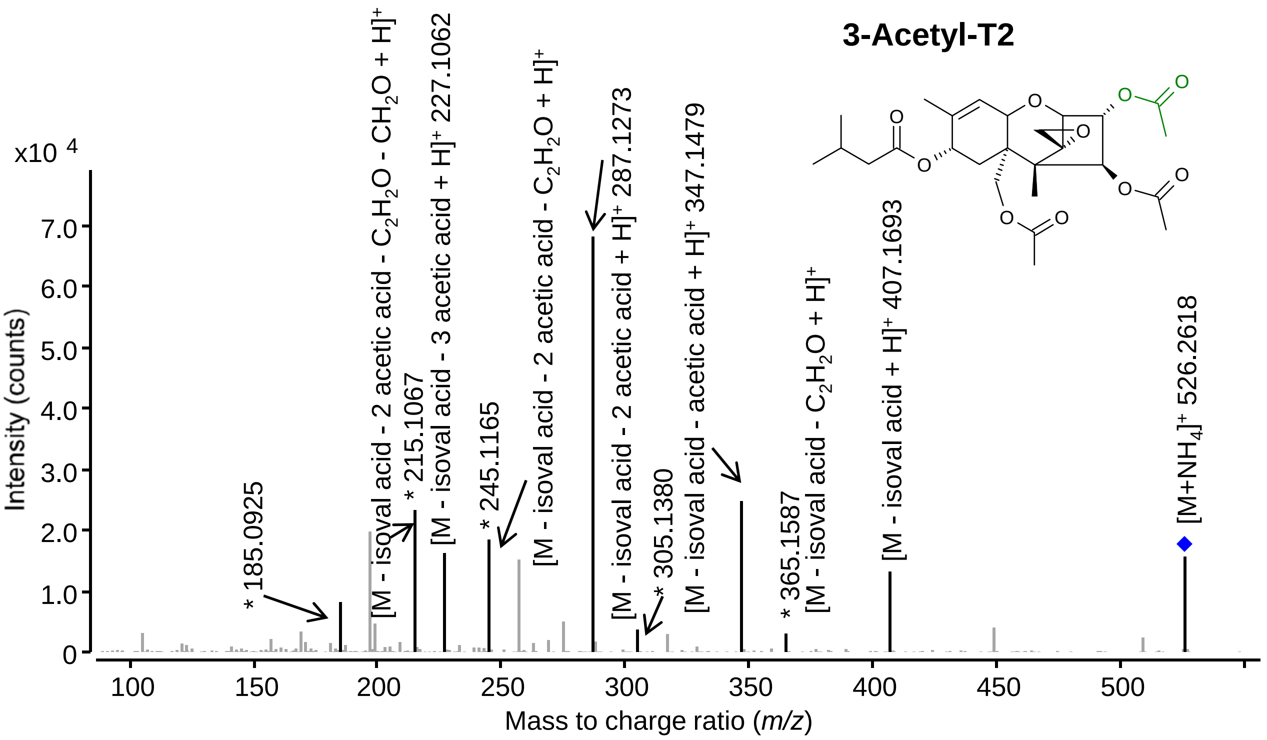

Fig. S11 LC-HRMS/MS-spectrum of 3-acetyl-T2, an *in planta* metabolite of T-2 toxin. Analysis was performed with a 6550 iFunnel Q-TOF LC/MS system in positive electrospray ionisation mode with a collision energy of 10 V. The ammonium adduct was chosen as precursor (marked with a diamond). Characteristic fragments used for annotation are highlighted and characteristic T-2 toxin fragments are marked with an asterisk (*). T2 (T-2 toxin), isoval acid (isovaleric acid)

Table S12 Signal suppression or enhancement (%) of different dilutions of 1 day and ripen barley extraction solution determined for HT2, T2, HT2-3-*O*-*β*-Glc, T2-Glc and 3-acetyl-T2.

Analyte	Undiluted		1+9		1+49	
	1 day	ripen	1 day	ripen	1 day	ripen
**HT2**	137±4	144±3	107±2	114±1	107±1	108±1
**T2**	116±5	106±3	102±3	102±1	104±1	103±1
**HT2-3-*O*-*β*-Glc**	138±3	142±2	106±1	112±3	107±1	106±1
**T2-Glc**	131±3	134±1	105±3	109±1	104±1	105±0.3
**3-Acetyl-T2**	89±3	88±3	98±4	98±1	104±1	102±1

HT2 (HT-2 toxin), T2 (T-2 toxin), Glc (glucoside). Mean value of spiked biological triplicate ± relative standard deviation is stated.
